# Laboratory measurements of the physics of auroral electron acceleration by Alfvén waves

**DOI:** 10.1038/s41467-021-23377-5

**Published:** 2021-06-07

**Authors:** J. W. R. Schroeder, G. G. Howes, C. A. Kletzing, F. Skiff, T. A. Carter, S. Vincena, S. Dorfman

**Affiliations:** 1 Department of Physics, Wheaton College, Wheaton, IL USA; 2 Department of Physics and Astronomy, University of Iowa, Iowa City, IA USA; 3 Department of Physics and Astronomy, University of California, Los Angeles, CA USA; 4 Space Science Institute, Los Angeles, CA USA

**Keywords:** Aurora, Plasma physics

## Abstract

While the aurora has attracted attention for millennia, important questions remain unanswered. Foremost is how auroral electrons are accelerated before colliding with the ionosphere and producing auroral light. Powerful Alfvén waves are often found traveling Earthward above auroras with sufficient energy to generate auroras, but there has been no direct measurement of the processes by which Alfvén waves transfer their energy to auroral electrons. Here, we show laboratory measurements of the resonant transfer of energy from Alfvén waves to electrons under conditions relevant to the auroral zone. Experiments are performed by launching Alfvén waves and simultaneously recording the electron velocity distribution. Numerical simulations and analytical theory support that the measured energy transfer process produces accelerated electrons capable of reaching auroral energies. The experiments, theory, and simulations demonstrate a clear causal relationship between Alfvén waves and accelerated electrons that directly cause auroras.

## Abstract (web summary)

It was predicted that Alfvén waves can account for the acceleration of precipitating auroral electrons. Here, the authors show laboratory measurements of the resonant transfer of energy from Alfvén waves to electrons under conditions relevant to the auroral zone as a direct test.

## Introduction

Space weather embodies the study of how variable forcing by the Sun, mediated by the supersonically flowing solar wind, affects the near-Earth space environment^1^. One of the most spectacular displays of the Sun’s effect on the Earth is the aurora, statistically appearing in an oval-shaped region around the magnetic poles at high latitude^2^, with manifestations on both the nightside and the dayside. High-energy particles precipitating down along the Earth’s dipolar magnetic field into the auroral ionopshere collisionally excite atoms and molecules^3^, leading to the auroral emissions with a variety of appearances, from bright discrete arcs, to faint arcs, to diffuse aurora. The differing magnetic local time and morphology of observed auroral events suggests distinct source regions as well as differing mechanisms of generation, with a clear disconnect between dayside and nightside aurora^4^. Three main magnetospheric drivers for the aurora have been identified^5^: (i) the precipitation of very energetic magnetosheath particles from the magnetospheric boundary layer on the dayside^6^ or plasma sheet electrons on the nightside^7,8^; (ii) quasi-static, field-aligned currents^9,10^; or (iii) energetic electrons accelerated by Alfvénic fluctuations, either as field-line resonances (effectively global-scale standing Alfvén waves in the Earth’s dipolar magnetic field)^11^ or Alfvén waves propagating down the field lines towards the auroral ionosphere^12,13^. For all of these cases, the detailed kinetic plasma physics governing the flow of energy from the outer magnetosphere into precipitating energetic particles remains a topic of ongoing study.

To confirm the acceleration of electrons by downward propagating Alfvén waves and assess their contribution to the total auroral emission, the space physics community has pursued a combination of spacecraft conjunction studies and statistical studies of the spatial distribution of Alfvén waves and precipitating electrons. Measurements from the Polar spacecraft at altitudes $3\,{\rm{{R}}_{E}}\, \lesssim\, z\, \lesssim\, 6\,{\rm{{R}}_{E}}$ (Earth radii) show that the downward Poynting flux of Alfvén waves was sufficient to power the intense auroral emission observed at magnetically conjugate points in the ionosphere^14,15^. Subsequent conjunction studies between Polar or Cluster at $z\, \gtrsim\, 4\,{\rm{{R}}_{E}}$ and FAST below at $z \sim 0.5\,{\rm{{R}}_{E}}$ showed a transition from downward Alfvén Poynting flux at high altitudes to significant precipitating energetic electron flux at lower altitudes^5,16,17^, suggesting that Alfvén wave energy is lost through the acceleration of electrons over the intervening altitude range. Although the electron acceleration is generally thought to occur over the altitude range $1\,{\rm{{R}}_{E}}\, \lesssim\, z\, \lesssim\, 2\,{\rm{{R}}_{E}}$^18,19^, sorting Alfvén Poynting flux by altitude shows a significant drop in amplitude over $3\,{\rm{{R}}_{E}}\, \lesssim\, z\, \lesssim\, 4\,{\rm{{R}}_{E}}$^20^. This fundamental picture of auroral electron acceleration by Alfvén waves also appears relevant to other planetary magnetospheres, with recent Juno measurements showing Alfvénic fluctuations associated with auroral emissions at Jupiter^21^.

Statistical studies of the spatial distribution of Alfvén wave power and precipitating electron flux using FAST and Polar measurements have shown that the spatial distribution in magnetic local time and latitude of Alfvén wave Poynting flux is well correlated with the distribution of accelerated electrons and the auroral emission^22–24^, with modeling suggesting the accelerated electrons primarily arrive from an altitude of $2\,{\rm{{R}}_{E}}\, \lesssim\, z\,\lesssim\, 3\,{\rm{{R}}_{E}}$^22^. Furthermore, the Alfvén wave Poynting flux was found to be sufficient to power ~1/3 of the global auroral luminosity, and up to 50% in the pre-midnight sector where substorm driven auroral events occur^23,24^, and Alfvén waves may be the dominant mechanism of electron acceleration during geomagnetic storms^20,25,26^.

Together, these conjunction and statistical studies build a strong case for the Alfvén-wave acceleration of auroral electrons. To develop a complete physical understanding of this critical element of magnetosphere-ionosphere coupling, it is essential to identify the kinetic plasma physics governing the acceleration of electrons by Alfvén waves. Analytical considerations and numerical modeling have framed the basic kinetic physics involved. Over the auroral acceleration region from $1\,{\rm{{R}}_{E}}\,\lesssim \,z\,\lesssim\, 3\,{\rm{{R}}_{E}}$, the Earth’s relatively high magnetic field *B*_0_ and low plasma electron density *n*_*e*_ and temperature *T*_*e*_ lead to conditions in the inertial regime, characterized by an Alfvén velocity greater than the electron thermal velocity, *v*_*A*_ > *v*_*te*_^27,28^. The Alfvén waves measured in the auroral acceleration region have typical length scales perpendicular to the magnetic field **B**_0_ that are comparable to the electron skin depth. Unlike the Alfvén waves of ideal MHD^29^, inertial Alfvén waves under these conditions are dispersive and give rise to an electric field component parallel to **B**_0_^28,30^. It is proposed that the parallel electric field of these waves can accelerate the precipitating electrons that power the aurora^13,28,31^. Kletzing^32^ mapped a distribution of test particles through prescribed inertial Alfvén wave fields and found electrons can be accelerated to auroral energies through a process similar to single-bounce Fermi acceleration where electrons gain energy while being overtaken by the wave and then are accelerated out of the front of the wave. Additional tests with realistic altitude profiles of the plasma parameters indicated that auroral electrons could be resonantly accelerated by inertial Alfvén waves^33,34^.

It is scientifically desirable to confirm definitively the kinetic physics of Alfvénic electron acceleration by simultaneously measuring the Alfvén wave fields and the resulting changes to the electron velocity distribution. Both measurements are essential to diagnose directly the transfer of energy between the waves and electrons. Spacecraft measurements, suffering uncertainty from plasma inhomogeneity, field line mapping, spacecraft motion, and limited points of measurement, have not been able to provide such a direct test.

In this work, we overcome the inherent limitations of spacecraft measurements by performing laboratory experiments^35^ on the Large Plasma Device (LAPD), a joint National Science Foundation/Department of Energy user facility at UCLA^36^. Inertial Alfvén waves are launched in the LAPD plasma and measurements of the electron velocity distribution are simultaneously collected using the absorption of a small amplitude whistler-mode wave. An analysis of measurements using the field-particle correlation technique gives a velocity-space signature indicating electrons are resonantly accelerated by inertial Alfvén waves in the experiment. Analytical kinetic theory, numerical Liouville mapping of a test particle distribution, and nonlinear gyrokinetic simulation are consistent with data from the experiment. The energy gain per electron per second in the experiment is consistent with an estimation using parameters from the auroral zone. While it was previously known that Alfvén waves are often coincident with auroras, this direct measurement demonstrates a causal relationship between Alfvén waves and accelerated electrons that produce auroras.

## Results

The LAPD is a 20 m long, 1 m diameter cylindrical vacuum chamber with a strong axial magnetic field of ${{\bf{B}}}_{0}=0.1700\hat{{\bf{z}}}$ T. While the characteristic parameters (magnetic field, density, and temperature) of the LAPD experiments differ by orders of magnitude from those in the auroral magnetosphere, a similarity analysis can be exploited to demonstrate that the key dimensionless parameters governing the physics of auroral electron acceleration can be reproduced in the laboratory^35,37^. The key condition determining the acceleration of auroral electrons is that the ratio of the electron thermal velocity to the Alfvén velocity is less than unity, *v*_*te*_/*v*_*A*_ < 1. Our experiments achieve a ratio *v*_*te*_/*v*_*A*_ = 0.35 such that the parallel resonant velocity of inertial Alfvén waves falls in the suprathermal tail of the electron distribution (Supplementary Methods 1).

### Alfvén wave measurements

**Fig. 1:**
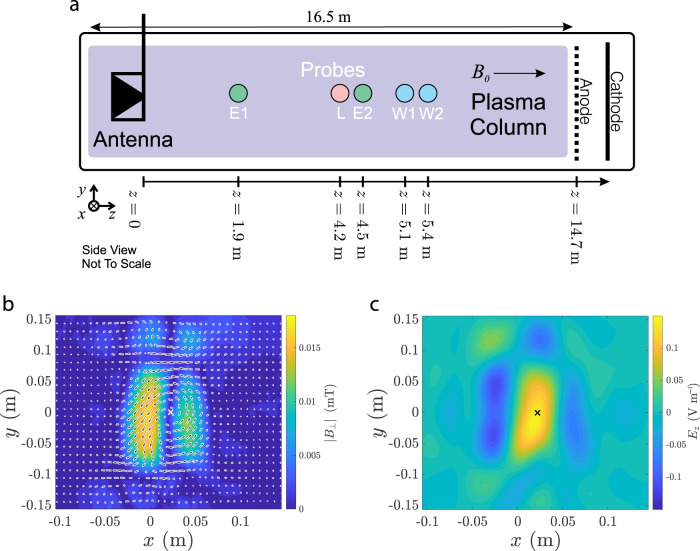
LAPD setup and snapshots of the Alfvén wave. **a** Experimental setup showing the Sigma antenna, Elsässer probes (E1, E2), Langmuir probe (L), and WWAD probes (W1, W2). **b** Contour plot of **B**_⊥_(*x*, *y*) of the inertial Alfvén wave in the plane perpendicular to **B**_0_ measured at E2. Arrows indicate the magnitude and direction of **B**_⊥_(*x*, *y*). **c** The calculated *E*_*z*_(*x*, *y*) midway between the WWAD probes W1 and W2. The *x* indicates the (*x*, *y*) location of the WWAD measurements of *g*_*e*_(*v*_*z*_).

We use the specially designed Sigma antenna to launch inertial Alfvén waves from one end of the chamber, as shown in Fig. 1a, Elsässer probes^38^ to record the electromagnetic fields, and a Whistler Wave Absorption Diagnostic (WWAD)^39^ to make simultaneous measurements of the parallel electron velocity distribution^40–43^ (see Methods for more detail on the experimental setup and procedure). The propagation of Alfvén waves between probes E1 and E2 is found to agree with the inertial Alfvén wave dispersion relation that includes the effects of thermal collisions (Supplementary Methods 2). In Fig. 1, over the plane (*x*, *y*) perpendicular to the axial magnetic field, we show (b) the measured Alfvén wave field **B**_⊥_ at E2 and (c) the predicted parallel electric field *E*_*z*_ at the WWAD, calculated using **B**_⊥_ and Ampére’s law (Supplementary Methods 2). The (*x*, *y*) position of the electron velocity distribution function measurement using the WWAD is indicated in Fig. 1b, c by the *x*, chosen at a maximum of the Alfvén wave *E*_*z*_, i.e. a current channel.

### Distribution function measurements

High-precision measurements of the suprathermal tails of the parallel electron velocity distribution *g*_*e*_(*v*_*z*_) were performed using the WWAD^39–43^. This measurement technique provides sufficient time resolution and precision to determine variations of *g*_*e*_(*v*_*z*_) as the Alfvén wave phase advances^44^ (Supplementary Methods 3). The wave absorption technique is unable to provide measurements of *g*_*e*_(*v*_*z*_) through the lower-velocity bulk of the distribution; however, because resonant acceleration affects suprathermal electrons, we are still able to explore this phenomenon with the available measurements (Supplementary Methods 3).

**Fig. 2:**
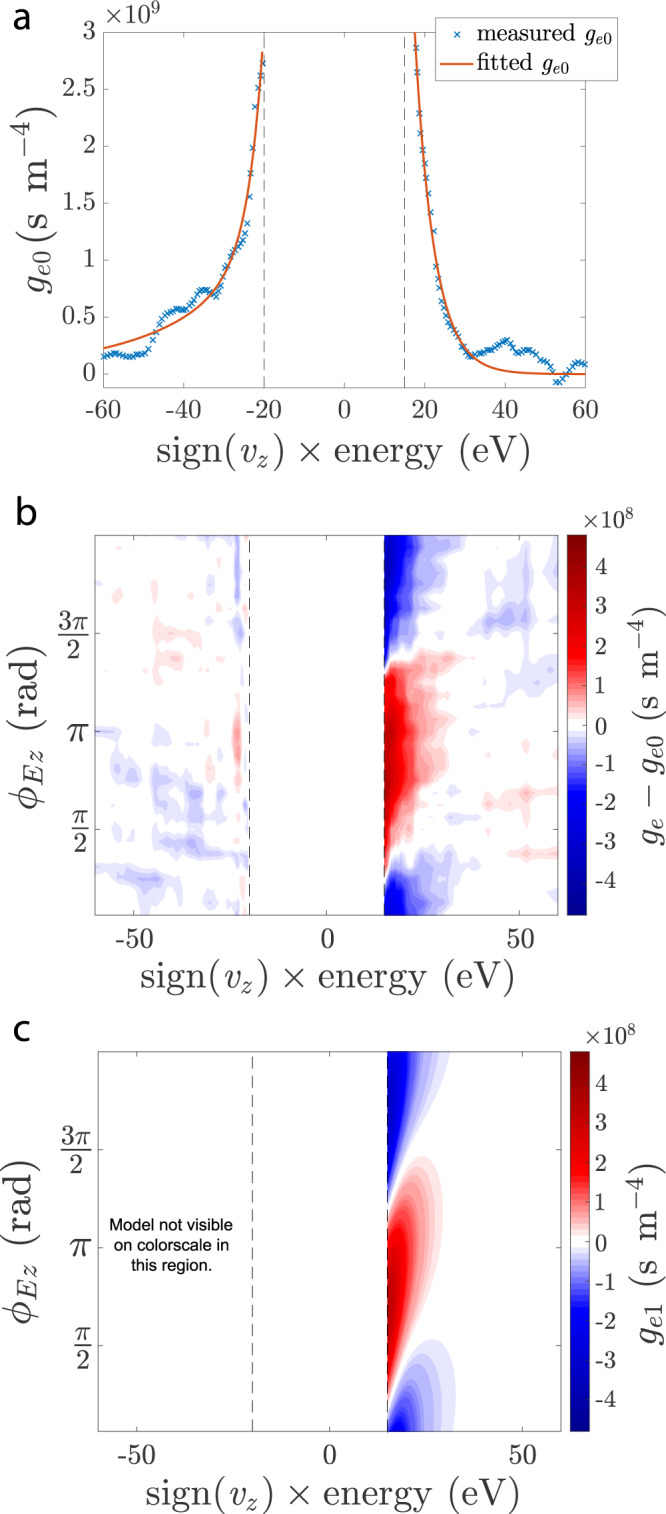
Measurements and model of the electron distribution. **a** The background distribution *g*_*e*0_(*v*_*z*_) obtained by averaging *g*_*e*_(*v*_*z*_) over Alfvén wave phase ${\phi }_{{E}_{z}}$ referenced to *E*_*z*_. **b** Measured perturbations *δg*_*e*_ are coherent with Alfvén wave phase ${\phi }_{{E}_{z}}$. **c** A solution to the linearized Boltzmann equation accurately models *g*_*e*1_ caused by Alfvén waves in this experiment. WWAD measurements of *g*_*e*_(*v*_*z*_) are unavailable in the region between the vertical dashed lines.

In Fig. 2a, we show the background distribution *g*_*e*0_(*v*_*z*_), obtained from a Fourier transform of distribution function measurements over the Alfvén wave period *T* = 2*π*/*ω*, where the contribution from the *n*th Fourier harmonic is given by *g*_*en*_(*v*_*z*_, *t*). Each side of *g*_*e*0_(*v*_*z*_) is fitted with a two-temperature Maxwellian distribution (red): a dominant, cold thermal core consistent with the Langmuir probe measurements of *T*_*e*_ and a hotter, diffuse component. The hotter 60 eV population seen for *v*_*z*_ < 0 corresponds to the energy of the primary electrons accelerated across the cathode-anode potential drop of the plasma source.

In Fig. 2b, we plot measurements of the total perturbation *δg*_*e*_(*v*_*z*_) = *g*_*e*_(*v*_*z*_) − *g*_*e*0_(*v*_*z*_) over 2*π* of Alfvén wave phase, showing a signal along the vertical axis dominated by the fundamental mode of oscillations *g*_*e*1_(*v*_*z*_) at the frequency *ω* of the Alfvén wave. A previously derived analytical solution to the linearized Boltzmann equation^44,45^, shown in Fig. 2c, reproduces the dominant features of this pattern using only *g*_*e*1_(*v*_*z*_). While the linear solution to the Boltzmann equation includes a velocity-dependent Coulomb collision rate, the typical suprathermal electron will travel nearly collisionlessly through the length of the experiment (Supplementary Methods 1). The upward slant of the blue and red features in the *v*_*z*_ > 0 region of Fig. 2b, c indicates phase variation in the response of *g*_*e*_(*v*_*z*_) to the Alfvén wave, i.e. faster electrons are not oscillating with the same phase relative to the Alfvén wave as slower electrons. As we will show, this difference in phase means that in some regions of velocity space the electrons gain energy, while in other regions they lose energy, ultimately producing a signature characteristic of resonant energy transfer^46–48^.

### Field-particle correlation of experimental data

Using single-point measurements of the electric field and electron velocity distribution, the rate of energization of electrons as a function of their velocity in the experiment is diagnosed using the field-particle correlation technique^46–48^ (Supplementary Methods 4). To determine the net rate of work done by *E*_*z*_ on the electrons, we use the measured *g*_*e*1_(*v*_*z*_, *t*) and *E*_*z*_(*t*) to compute the parallel field-particle correlation, defined by

1 $${C}_{{E}_{z}}({v}_{z})={\rm{Corr}}\ \left(\frac{e{v}_{z}^{2}}{2}\frac{\partial {g}_{e1}({v}_{z},t)}{\partial {v}_{z}},{E}_{z}(t)\right),$$

where this unnormalized correlation is an average of the product of the terms over one full Alfvén wave period. This correlation ${C}_{{E}_{z}}({v}_{z})$ yields the rate of change of parallel phase-space energy density, ${w}_{e}({v}_{z})={m}_{e}{v}_{z}^{2}{g}_{e}({v}_{z})/2$, as a function of the parallel velocity *v*_*z*_ attributable to the interaction of electrons with the Alfvén wave field *E*_*z*_. Key features of the field-particle correlation as a function of *v*_*z*_, such as regions where ${C}_{{E}_{z}}({v}_{z})$ is positive or negative as well as the location of zero crossings, are referred to collectively as the velocity-space signature, and can be compared to the velocity-space signatures of known wave-particle interactions.

**Fig. 3:**
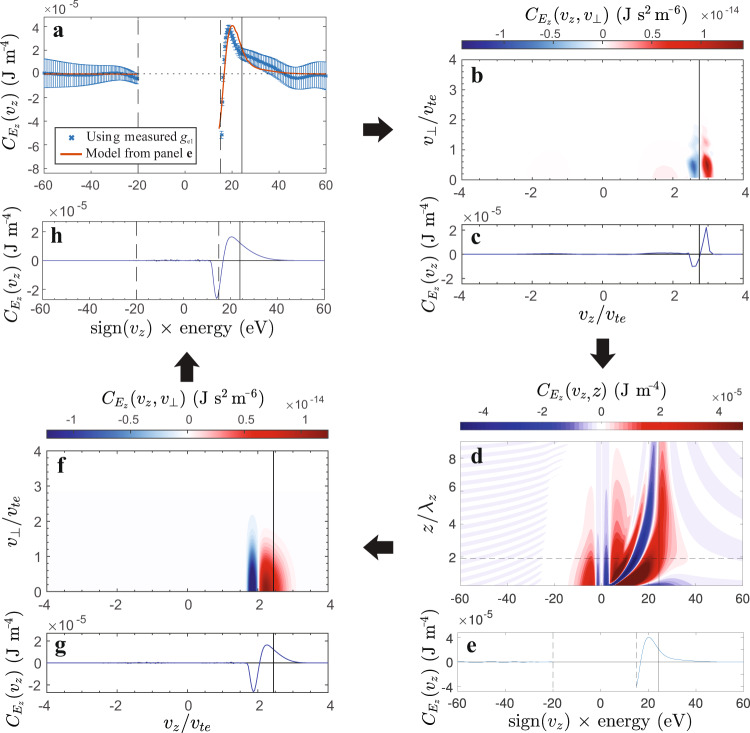
Field-particle correlations ${C}_{{E}_{z}}({v}_{z})$ and ${C}_{{E}_{z}}({v}_{z},{v}_{\perp })$. Results from **a** LAPD experiment, **b**, **c** AstroGK simulation, **d**, **e** an analytical Laplace-Fourier Transform solution of the *z*/*λ* evolution of ${C}_{{E}_{z}}({v}_{z})$, and **g**, **f**, **h** Liouville mapping. **h** is a re-plotting of (**g**) with axes for direct comparison with (**a**). Gyrotropic correlations ${C}_{{E}_{z}}({v}_{z},{v}_{\perp })$ in (**b**, **f**) are reduced to the parallel correlations ${C}_{{E}_{z}}({v}_{z})$ in (**c**, **g**) by integration over *v*_⊥_. The parallel phase velocity *v*_*ph*_ is noted by a black vertical line on all plots. WWAD measurements are unavailable in the region bounded by vertical dashed lines. The dashed horizontal line in (**d**) identifies the slice re-plotted in (**e**, **a**) and corresponds to the *z*/*λ*_*z*_ location of WWAD measurements in the experiment. The AstroGK ${C}_{{E}_{z}}({v}_{z})$ (**c**), with a bipolar signature centered on *v*_*ph*_, is comparable to the largest values of *z*/*λ*_*z*_ in (**d**). The analytical (**e**) and Liouville mapping (**g**) ${C}_{{E}_{z}}({v}_{z})$ include finite *z*/*λ* of the experiment and produce a bipolar signature below *v*_*ph*_ similar to (**a**) the experimental ${C}_{{E}_{z}}({v}_{z})$. Error bars in (**a**) represent the standard deviation. The black arrows represent the path of analytical and numerical analysis leading to closure in the interpretation of our experimental measurements.

Applying the field-particle correlation to the measured *g*_*e*1_(*v*_*z*_) gives ${C}_{{E}_{z}}({v}_{z})$ shown in Fig. 3a, where the vertical line around 24 eV shows the energy corresponding to the parallel wave phase velocity *v*_*z*_ = *v*_*ph*_. The transition of ${C}_{{E}_{z}}({v}_{z})$ from negative to positive indicates the energy density of the electron distribution is decreasing at lower velocities and increasing at higher velocities. Changes in the phase-space energy density *w*_*e*_(*v*_*z*_) occur only when the number of particles at a given velocity *v*_*z*_ changes. For example, when electrons are accelerated to a higher velocity, *w*_*e*_(*v*_*z*_) decreases at their initial lower velocity and increases at their final higher velocity. This produces a bipolar signature in ${C}_{{E}_{z}}({v}_{z})$—negative at the lower velocity, and then crossing through zero and becoming positive at the higher velocity. Using this insight to provide a physical interpretation of Fig. 3a, electrons are being moved within the distribution from lower velocity to higher velocity by the Alfvén wave. This is direct experimental evidence for the acceleration of electrons by inertial Alfvén waves. For help identifying the wave-particle interaction responsible for the electron acceleration detected experimentally, we turn to analytical theory and numerical simulation.

### Comparison to numerical simulations and analytical theory

To interpret the experimental ${C}_{{E}_{z}}({v}_{z})$, we begin by comparing the experimental ${C}_{{E}_{z}}({v}_{z})$ with a nonlinear gyrokinetic simulation of inertial Alfvén waves (Supplementary Methods 5). The electromagnetic fields and velocity distributions of ions and electrons of a simulated plasma are self-consistently advanced assuming the distributions are gyrotropic, or particles are performing uniform circular motion around the magnetic field. Using plasma parameters relevant to the experiment, we initialize an inertial Alfvén wave in a periodic domain and use the resulting electron velocity distribution and *E*_*z*_(*t*) of the inertial Alfvén wave at a single point to compute ${C}_{{E}_{z}}$ in Fig. 3b shown in gyrotropic velocity space (*v*_*z*_, *v*_⊥_) and (c) reduced to *v*_*z*_. Gyrokinetic results are presented in the typical way with axes showing velocity normalized to the electron thermal speed. The gyrokinetic ${C}_{{E}_{z}}({v}_{z})$, bipolar with a zero crossing at *v*_*ph*_, is the known velocity-space signature of electron acceleration via Landau damping^48^. This wave-particle interaction can be interpreted physically as electrons being accelerated by the wave to velocities greater than the wave itself, similar to how a surfer can end up traveling faster than the crest of the ocean wave. The gyrokinetic result is not unexpected since test particle simulations have predicted inertial Alfvén waves accelerate electrons via Landau resonance^32–34^.

Although gyrokinetic results in Fig. 3b, c are presented using velocity axes conventional for gyrokinetic simulation while experimental results in (a) are plotted using an energy axis, key similarities and differences are evident. Like the gyrokinetic result, the experimental ${C}_{{E}_{z}}({v}_{z})$ is also bipolar. Unlike the gyrokinetic result, the zero crossing of the experimental ${C}_{{E}_{z}}({v}_{z})$ falls below *v*_*ph*_. This discrepancy between the zero crossing of the experimental ${C}_{{E}_{z}}({v}_{z})$ and the wave phase velocity is explained by accounting for the finite distance of the experimental measurement from the antenna. To model this effect, we have analytically solved the linearized Boltzmann equation using a Laplace transform for an inertial Alfvén wave generated at *z* = 0 (Supplementary Methods 6). The analytical solution allows us to predict in Fig. 3d how ${C}_{{E}_{z}}({v}_{z})$ varies as a function of distance *z* from the Alfvén wave antenna, where the vertical axis is normalized by the parallel wavelength, *z*/*λ*_*z*_. Figure 3d helps interpret both the gyrokinetic and the experimental ${C}_{{E}_{z}}({v}_{z})$. With its periodic domain, the gyrokinetic simulation in (c) effectively has a long interaction length, making it most comparable to the largest values of *z*/*λ*_*z*_ in (d), where indeed we find that the zero crossing of the velocity-space signature falls at *v*_*ph*_. Conversely, the experimental ${C}_{{E}_{z}}({v}_{z})$ in (a) is measured near *z*/*λ*_*z*_ ≃ 2, and we see in (d) that the zero crossing of the velocity-space signature at that position is predicted to fall below the phase velocity, as observed in the experiment. A slice along *z*/*λ*_*z*_ ≃ 2 (dashed horizontal line) in (d) is plotted in (e), showing that, near the antenna, the zero crossing of ${C}_{{E}_{z}}({v}_{z})$ occurs at a velocity below the wave phase velocity, *v*_*z*_ < *v*_*ph*_. Note that the data in panel (e) is the same data as the model results (red) plotted in panel (a), so the theoretical prediction of ${C}_{{E}_{z}}({v}_{z})$ yields quantitative agreement with the experiment. Physically, the signature of the zero crossing of ${C}_{{E}_{z}}({v}_{z})$ falling below the resonant velocity is due to electrons originating at *z* = 0 that start below *v*_*ph*_ being resonantly accelerated but having not yet reached their maximum velocity *v*_*z*_ ≳ *v*_*ph*_. Together, these results form a consistent picture that the velocity-space signature arising from the Landau resonance evolves as resonant electron acceleration unfolds over a finite distance *z* from the antenna.

While the gyrokinetic simulation is not well-suited to finite interaction lengths, a mapping of phase space through the predicted wave fields according to Liouville’s theorem is an established technique^32,33^ that is capable of modeling finite length interactions. Liouville mapping provides an independent approach to determine if indeed the zero crossing of ${C}_{{E}_{z}}({v}_{z})$ falls below *v*_*ph*_ as suggested by analytical theory in Fig. 3d when the distance from the antenna is *z*/*λ*_*z*_ ≃ 2. In Fig. 3f, we present the gyrotropic field-particle correlation from such a Liouville mapping (Supplementary Methods 7), and there are signs of agreement with the experimental ${C}_{{E}_{z}}({v}_{z})$. In (f), we see the expected bipolar velocity-space signature characteristic of the Landau resonant acceleration, but, in agreement with the analytical prediction, the zero crossing falls below *v*_*ph*_ due to the finite distance *z*/*λ*_*z*_ ≃ 2 from the antenna. Integrating over *v*_⊥_ yields (g) a reduced parallel velocity-space signature that makes clear these distinguishing features. To enable a more direct comparison to the experimental results, we convert the horizontal axis of (g) from parallel velocity *v*_*z*_ to the associated energy, as shown in Fig. 3h. This final Liouville mapping prediction shows qualitative agreement with our experimental determination of ${C}_{{E}_{z}}({v}_{z})$.

The discrepancy in magnitude between Fig. 3a and h is attributed largely to the fact that Liouville mapping includes only the dominant plane wave mode present in the launched Alfvén wave in Fig. 1, neglecting all harmonics present in the experiment (Supplementary Methods 8). But, the quantitative agreement of the zero crossing in the bipolar velocity-space signature of ${C}_{{E}_{z}}({v}_{z})$ between (a) the experiment and (h) the Liouville mapping model definitively identifies the Landau resonance as the mechanism of electron acceleration. The discrepancy in magnitude does not suggest an alternate acceleration mechanism is at work. The agreement between the experimentally determined ${C}_{{E}_{z}}({v}_{z})$ and the various model results confirm that we have experimentally identified resonant energy transfer from inertial Alfvén waves to electrons.

### Connection to an accelerated beam of electrons

**Fig. 4:**
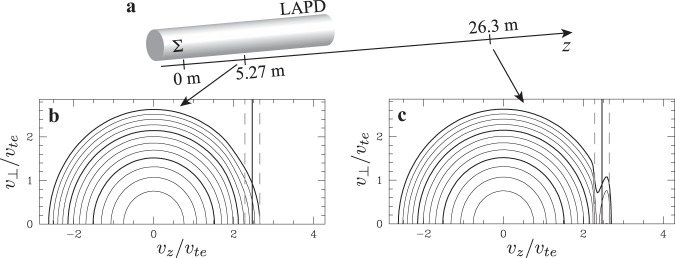
Liouville mapping over an extended region. **a** The Liouville mapping domain is extended beyond the length of the LAPD. Logarithmically spaced contours of the electron velocity distribution predicted by Liouville mapping **b** at the point of measurement at *z* = 5.27 m and **c** at a point five times further away (beyond the length of the LAPD experimental chamber) at *z* = 26.3 m, showing that with a longer interaction distance, a clear accelerated beam of electrons is visible. The vertical solid black line in (**b**, **c**) represents the resonant velocity. Vertical dashed lines indicate the nonlinear trapping width.

The measurements presented above demonstrate a resonant energy transfer rate between inertial Alfvén waves and electrons. Over an extended interaction region like the auroral zone, the cumulative effect of this energy transfer is a population of accelerated electrons. For the relatively short distance of interaction between the antenna and the point of measurement in the experiment (about two wavelengths), the resulting component of accelerated electrons may not be easily discernible. By extending the distance of the Liouville mapping calculation, we show that the experimentally detected energy transfer rate applied over a longer interaction region does indeed generate an accelerated population of electrons (Supplementary Methods 7). In Fig. 4, we plot the predicted gyrotropic electron velocity distribution (a) at the point of measurement at *z* = 5.27 m, or *z*/*λ*_*z*_ ≃ 2, and (b) at a distance five times further (over 10 m beyond the end of the LAPD experimental chamber) at *z* = 26.3 m, *z*/*λ*_*z*_ ≃ 10. Although the effect of the acceleration of electrons by the inertial Alfvén wave is difficult to discern at *z*/*λ*_*z*_ ≃ 2 by looking at the distribution function alone, by the time the wave reaches *z*/*λ*_*z*_ ≃ 10, the population of accelerated electrons would be clearly observed, with an accelerated beam reminiscent of early auroral acceleration modeling^32^. The resonant velocity (black solid) and nonlinear trapping width^49^ for the experimental wave amplitude (gray dashed) are indicated, showing a significant net acceleration of electrons within the nonlinear trapping width, as expected theoretically.

While measuring an accelerated population of electrons experimentally, for example by detecting a beam in the electron distribution far from the antenna, would provide supporting evidence for our conclusions, this measurement alone would not be sufficient to prove that we have observed electrons accelerated by an inertial Alfvén wave. A population of accelerated electrons can be produced by a number of nonlinear effects that may arise while launching high-power waves in laboratory plasmas. Conversely, the field-particle correlation allows the specific conclusion that energy is being transferred to electrons from the Alfvén wave, and the bipolar signature and its zero crossing make clear the responsible acceleration mechanism is the Landau resonance.

### Connection to the auroral acceleration region

We may compare the rate of energization of electrons by the inertial Alfvén wave in this experiment with a comparable case in the auroral acceleration region of the magnetosphere. The number density of accelerated electrons *n*_*acc*_ is computed by directly integrating the equilibrium distribution function *g*_*e*0_(*v*_*z*_) over the range of velocities where significant energization occurs, from *v*_*z*,*min*_/*v*_*te*_ = 1.96 (corresponding to our lower cutoff at 15.5 eV) to *v*_*z*,*max*_/*v*_*te*_ = 3.18 (or 40.6 eV), yielding *n*_*acc*_ = 1.59 × 10^15^ m^−3^. Similarly, integrating the parallel field-particle correlation ${C}_{{E}_{z}}({v}_{z})$ over the same velocity limits yields the net rate of energy transfer to these accelerated electrons (*dE*/*dt*)_*acc*_ = 20.6 J m^−3^ s^−1^. To find the average rate of energization per electron, we can divide these two numbers to obtain an energization rate of 8.1 × 10^4^ eV s^−1^ per electron.

**Fig. 5:**
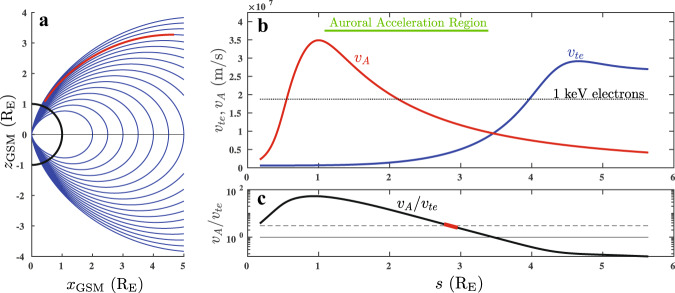
Model of the auroral zone. **a** Plot of the Earth’s dipole magnetic field lines (blue) and the segment (red) along the field line through the auroral acceleration region for *L* = 8.5 (or invariant latitude Λ = 70^∘^). Profiles of (**b**) the Alfvén speed *v*_*A*_ (red) and electron thermal velocity *v*_*te*_ (blue) as well as (**c**) their ratio *v*_*A*_/*v*_*te*_ (black) as a function of distance along the field line *s*. The dashed line shows the ratio *v*_*A*_/*v*_*te*_ ≃ 3 relevant to the LAPD experiments reported here. The highlighted red region shows the experimental *v*_*A*_/*v*_*te*_ corresponds to *v*_*A*_/*v*_*te*_ in the auroral zone near a distance *s* of 3 ${\rm{{R}}_{E}}$.

To make a meaningful comparison to the rate of energization per electron in the auroral acceleration region, we have developed a model (Supplementary Methods 9) of the plasma parameters as a function of distance along a magnetic field line given by *L* = 8.5, or invariant latitude *λ* = 70^∘^. In Fig. 5, we show (a) the inner magnetosphere’s dipolar magnetic field lines (blue) in Geocentric Solar Magnetospheric coordinates, and we highlight (red) the segment along the *L* = 8.5 field line from an altitude *z* = 1000 km. We also show (b) the profiles of Alfvén velocity *v*_*A*_ (red) and electron thermal velocity *v*_*te*_ (blue) vs. distance along the field line *s* from the Earth’s surface (measured in units of the Earth radii, $\,{\rm{{R}}_{E}}$). We also plot (c) the ratio *v*_*A*_/*v*_*te*_ vs. *s*, where the region corresponding to our experimental parameters with *v*_*A*_/*v*_*te*_ ≃ 3 is highlighted (red).

During magnetospheric substorms, when Alfvén waves are believed to drive a significant fraction of the observed discrete auroral arcs^24^, shifts in the magnetic field within the distant magnetotail (possibly due to magnetotail reconnection) are transmitted Earthward along the magnetic field as Alfvén waves^12,28^. In Fig. 5b, at distances along the field line $s\, \gtrsim\, 3.5\,{\rm{{R}}_{E}}$, the plasma is in the regime of kinetic Alfvén waves where *v*_*te*_ > *v*_*A*_, and therefore the parallel electron velocity that is resonant with the parallel phase velocity of Alfvén and kinetic Alfvén waves falls within the core of the electron velocity distribution at *v*_*z*_ < *v*_*te*_. In this case, resonantly damped wave energy is likely to be shared among the large population of these core electrons, and so the resonant interaction may lead to a broadening of the velocity distribution in the parallel direction, but may not be as effective in accelerating electrons to high energies. It should be mentioned, however, that several numerical studies have suggested that trapping of electrons in finite-amplitude kinetic Alfvén waves or kinetic-scale field-line resonances may be effective at accelerating electrons to keV energies^50–52^ in this regime at altitudes $z\, > \, 4\,{\rm{{R}}_{E}}$.

At distances $s\, \lesssim\, 3.5\,{\rm{{R}}_{E}}$ in Fig. 5b, the plasma is in the regime of inertial Alfvén waves where *v*_*A*_ > *v*_*te*_, so the resonant parallel electron velocity falls within the suprathermal tail of the electron velocity distribution, *v*_*z*_ > *v*_*te*_. In this case, the resonantly damped wave energy is shared by only the small population of electrons in the tail, and these electrons may be more effectively accelerated to higher energies. In fact, since the Alfvén wave velocity is observed to increase as *s* decreases over the range $1\,{\rm{{R}}_{E}}\, \lesssim\, s\,\lesssim\, 3.5\,{\rm{{R}}_{E}}$, accelerated electrons can stay resonant with these accelerating Alfvén waves as they propagate toward the Earth, enabling these electrons to reach keV energies (where the horizontal dotted line indicates the parallel velocity of an electron with 1 keV of kinetic energy)^32,33^. It is this range of distances along the field line, which corresponds in our model to altitudes $1\,{\rm{{R}}_{E}}\, \lesssim\, z\,\lesssim\, 3\,{\rm{{R}}_{E}}$, that is traditionally denoted the auroral acceleration region (green horizontal line)^53^.

To compare the average rate of energization of resonant electrons in the auroral acceleration region, we use the plasma parameters from the auroral acceleration region model (Supplementary Methods 9) to compute the number density of acceleration electrons *n*_*acc*_ and the rate of change of energy density (*dE*/*dt*)_*acc*_ at the comparable point (with *v*_*A*_/*v*_*te*_ ≃ 3) along the field line at $s\simeq 2.85\,{\rm{{R}}_{E}}$, corresponding to an altitude $z\simeq 2.56\,{\rm{{R}}_{E}}$. At this point, the model yields plasma parameters *B*_0_ = 1140 nT, *n*_*e*_ = 4 × 10^6^ m^−3^, and *T*_*e*_ = 50 eV. The number density of electrons that are resonant with the Alfvén wave are estimated by integrating a Maxwellian electron velocity distribution over the same normalized limits as the experiment, *v*_*z*,*min*_/*v*_*te*_ = 1.96 and *v*_*z*,*max*_/*v*_*te*_ = 3.18. We obtain *n*_*acc*_ ≃ *n*_*e*_ erfc(1.96) = 0.0036*n*_*e*_ = 1.44 × 10^4^ m^−3^, where erfc denotes the complementary error function.

The rate of transfer of energy density from the inertial Alfvén wave to these resonant electrons is estimated using the linear collisionless damping rate *γ* and wave magnetic energy density for these parameters, ${(dE/dt)}_{acc}=2\gamma {(\delta {B}_{\perp })}^{2}/2{\mu }_{0}$. For the plasma parameters *v*_*A*_/*v*_*te*_ = 3 and *T*_*i*_/*T*_*e*_ = 1, the linear Vlasov-Maxwell dispersion relation^54,55^ yields a normalized complex wave frequency *ω*/*k*_∥_*v*_*A*_ = (0.795, − 1.5 × 10^−2^) for a wave with *k*_⊥_*δ*_*e*_ = 1, the typical perpendicular scale where the inertial Alfvén wave parallel electric field leads to resonant energization via the Landau resonance. For an estimated parallel wavelength of *L*_∥_ ~ 500 km and a wave amplitude at this altitude of *δB*_⊥_ ~ 10 nT, we obtain a energization rate of (*dE*/*dt*)_*acc*_ = 1.9 × 10^−10^ J m^−3^ s^−1^. Dividing these two numbers to obtain the average rate of energization per electron, we obtain 8.2 × 10^4^ eV s^−1^ per electron. Uncertainty in our estimated value of the parallel wavelength *L*_∥_ and the use of an idealized Maxwellian equilibrium velocity distribution mean that this energization rate is only an order-of-magnitude estimate, but this value is clearly consistent with that found in the experiment. By designing a scaled laboratory experiment to obtain the same key dimensionless parameter *v*_*A*_/*v*_*te*_ ≃ 3, we have shown that the energization rate per electron by the inertial Alfvén wave agrees with the prediction for the auroral acceleration region.

## Discussion

Early calculations by Kletzing^32,33^ predicted that, for realistic auroral acceleration region parameters, Alfvén wave acceleration can account for the quantitative features of precipitating auroral electrons. While this hypothesis is consistent with spacecraft measurements in auroral regions, it has not previously been directly tested. In this experiment, measured changes to the electron velocity distribution, when correlated with the Alfvén wave fields, indicate resonant electron acceleration. The same acceleration process is seen while modeling inertial Alfvén wave-particle interactions with gyrokinetic simulation, analytical theory, and Liouville mapping. Experimental results are reproduced by analytical theory and Liouville mapping once the finite length of the experiment is included. The experimental field-particle correlation, and its agreement with analytical theory and Liouville mapping, allow the specific conclusion that the inertial Alfvén wave in this experiment is accelerating electrons and that the responsible acceleration mechanism is Landau resonance. By extending Liouville mapping calculations over a longer interaction length, we show that the cumulative effect of the energy transfer detected in this experiment is the production of a distinct population of accelerated electrons. The agreement of the rate of energization per electron between the experiment and an auroral model at altitude $z \sim 2.5\,{\rm{{R}}_{E}}$ establishes the final connection needed to show we have provided direct experimental confirmation that Alfvén waves can accelerate electrons that precipitate into the ionosphere and generate the fascinating glow of the aurora.

## Methods

The LAPD chamber is backfilled with H_2_ to 10^−5^ Torr. A heated nickel cathode, coated with barium oxide to lower the work function, emits primary electrons from one end of the vacuum vessel, shown in Fig. 1a, that collisionally dissociate and ionize the H_2_ fill gas, producing a plasma with electron density *n*_*e*_ = (1.2 ± 0.2) × 10^18^ m^−3^ and *T*_*e*_ = 4 ± 1 eV. Electron density and temperature are determined using a swept Langmuir probe, and absolute calibration of the density measurement is obtained using a microwave interferometer. Measured experimental parameters, derived characteristic plasma scales, and key dimensionless quantities of the experiment are presented in Supplementary Table 1. The LAPD produces a 10 ms shot of plasma every second. Data from several shots show the plasma is reproducible from shot to shot.

During each shot, a burst of traveling inertial Alfvén waves is launched at a fixed moment in the discharge sequence by our Sigma antenna at a frequency of 1.177 MHz. The Alfvén wave fields **B**_⊥_ and **E**_⊥_, perpendicular to **B**_0_, are recorded using Elsässer probes^38^ at a distance of $1.9\hat{{\bf{z}}}$ and $4.5\hat{{\bf{z}}}$ m from the Alfvén wave antenna. During each shot, each Elsässer probe provides measurements at a single location (*x*, *y*, *z*). Shot-to-shot fluctuations of the plasma conditions and Alfvén wave fields are normally distributed, and 10 shots of Elsässer probe data at a given location provides a sufficient signal-to-noise ratio to record the local temporal evolution of **B**_⊥_ and **E**_⊥_. Elsässer probes are mounted to an automated motion system so that 10 shots of data are collected at each point of a user-defined grid in the *x*–*y* plane. Additional properties of the Sigma antenna and the resulting inertial Alfvén waves are given in Supplementary Methods 2.

The WWAD^39^ provides high-precision measurements of the suprathermal tails of the reduced parallel electron velocity distribution *g*_*e*_(*v*_*z*_) at an approximately localized position in configuration space. The diagnostic is implemented with 1" dipole antennas, denoted as W1 and W2 in Fig. 1a, that are used as a transmitter and receiver separated by ${{\Delta }}z=0.3\hat{{\bf{z}}}$ m. Because *g*_*e*_(*v*_*z*_) is measured using the absorption of whistler waves between the dipole antennas, the measured *g*_*e*_(*v*_*z*_) is an average over the distance separating the antennas. However, the spacing of the WWAD antennas is much less than the Alfvén parallel wavelength, Δ*z*/*λ*_*z*_ = 0.12, so the measured *g*_*e*_(*v*_*z*_) is approximately local compared to the Alfvén wave. Measurements of *g*_*e*_(*v*_*z*_) are performed near (*x*, *y*, *z*) = (0.02, 0.00, 5.25) m. The *x* and *y* coordinates, marked by an *x* in Fig. 1b, c, were chosen because they are revealed by Elsässer probe measurements to be a maximum of **E**_*z*_, and therefore this location is also a maximum of the perturbation to *g*_*e*_(*v*_*z*_) caused by the Alfvén wave.

The WWAD measures *g*_*e*_(*v*_*z*_) with 10 μs resolution. To resolve the 0.85 μs period of the Alfvén wave, a series of 32 phase-shifted data sets is collected, where each data set consists of 1024 shots. Before each data set, the launch of the Alfvén wave burst is delayed by an additional 1/32 of the Alfvén wave period. Every time step in the measurement of *g*_*e*_(*v*_*z*_) is sorted into a grid indexed by electron velocity *v*_*z*_ and ${\phi }_{{E}_{z}}$ the phase of the Alfvén wave’s parallel electric field *E*_*z*_. Alfvén wave phase is determined from Elsässer probe measurements of the Alfvén wave fields detailed in Supplementary Methods 2. Additional information about measuring *g*_*e*_(*v*_*z*_) using the WWAD is provided in Supplementary Methods 3.

## Supplementary information

Supplementary Information

## Data Availability

The numerical simulations presented here are available from the corresponding author upon reasonable request. LAPD data are managed by the Basic Plasma Science Facility at UCLA (https://plasma.physics.ucla.edu) and are available from the Facility upon reasonable request.

## Code availability

All relevant AstroGK code and Matlab routines are available from the corresponding author upon reasonable request. AstroGK source code is available from https://bitbucket.org/gyrokinetics/.
