# Supplementary material for: Laboratory measurements of the physics of auroral electron acceleration by Alfvén waves
Source: Nat Commun. 2021 Jun 7;12:3103. doi: 10.1038/s41467-021-23377-5 (PMC8184961; doi:10.1038/s41467-021-23377-5)
Supplement: Supplementary file 1 — Supplementary Information [file 41467_2021_23377_MOESM1_ESM.pdf]

Supplementary information for

# Laboratory measurements of the physics of auroral electron acceleration by Alfvén waves

J. W. R. Schroeder[1], G. G. Howes[2], C. A. Kletzing[2], F. Skiff[2], T. A. Carter[3], S. Vincena[3], and S. Dorfman[4]

April 12, 2021

[1]Department of Physics, Wheaton College, Wheaton, Illinois 60187, USA.
[2]Department of Physics and Astronomy, University of Iowa, Iowa City, Iowa 52242, USA.
[3]Department of Physics and Astronomy, University of California, Los Angeles, California 90095-1547, USA.
[4]Space Science Institute, Los Angeles, California 90095-1547, USA.

**Supplementary Methods 1: Reproducing Auroral Magnetospheric Conditions in the Laboratory.**

While absolute parameters of the LAPD vary dramatically from those of the auroral magnetosphere [1], the experimental values of $\mathbf{B}_0$, $n_e$, and $T_e$ were chosen so that magnetic pressure dominates over plasma thermal pressure as it does in the auroral magnetosphere. Specifically, the plasma parameters in the acceleration region of the auroral magnetosphere lead to an electron plasma beta that is less than the electron-to-ion mass ratio, $\beta_e < m_e/m_i$. Under these extremely low-$\beta_e$ conditions, the electron thermal velocity is slower than the Alfvén velocity, which can be expressed by $v_{te}/v_A = (\beta_e m_i/m_e)^{1/2} < 1$, and so any resonant acceleration of electrons by the Alfvén waves is expected to occur in the suprathermal tail of the electron velocity distribution. The experiments presented here were performed in hydrogen plasma with $m_i/m_e = 1836$ and electron plasma beta $\beta_e = 6.69 \times 10^{-5}$, leading to the ratio of the electron thermal to the Alfvén velocity of $v_{te}/v_A = 0.35$, as shown in Supplementary Table 1.

Thermal electrons in the LAPD are much more collisional than those of the auroral magnetosphere, with a Braginskii thermal electron collision rate of $\nu_{te} = 4.4 \times 10^6$ s$^{-1}$. Through the linear dispersion relation for inertial Alfvén waves that includes the effect of collisions [2, 3], given by Supplementary Equation (1), a non-zero collision rate causes an increase in Alfvén wave damping and a slight decrease in the wave frequency. In our experiment, the ratio of the thermal electron collision frequency to the angular frequency of the Alfvén waves is $\nu_{te}/\omega = 0.6$, indicating that the electrons in the thermal core of the distribution are moderately collisional. Despite the effects of thermal collisions on the inertial Alfvén wave propagation and damping, the electrons that are resonantly accelerated by $E_z$ of the inertial Alfvén wave have resonant parallel velocities well above the electron thermal velocity, $v_r/v_{te} = \omega/k_z v_{te} > 1$, and therefore these suprathermal electrons are much less strongly affected by Coulomb collisions. The collision frequency is inversely proportional to the third power of the particle velocity, so the effective collision frequency for resonant electrons relative to the thermal electron collision frequency scales as $\nu_{re}/\nu_{te} = (v_{te}/v_r)^3$. For the inertial Alfvén waves in this experiment, we calculate that $\omega/k_z v_{te} = v_r/v_{te} = 2.4$, giving a normalized collision frequency for the resonant electrons of $\nu_{re}/\omega = 0.04$, so that the effect of collisions in the LAPD on the electron velocity distribution $g_{e1}(v_z)$ at the resonant velocity is minimal.

**Supplementary Methods 2: Measuring the Electromagnetic Fields of the Inertial Alfvén Wave.**

| Measured Quantity | |
|---|---|
| Axial Magnetic Field, $B_0$ | 0.17 T |
| Electron Density, $n_{e0}$ | $(1.2 \pm 0.2) \times 10^{18}$ m$^{-3}$ |
| Electron Temperature, $T_e$ | $4 \pm 1$ eV |
| Ion Temperature, $T_i$ | $1^{+1}_{-0.5}$ eV |
| Sigma Antenna Frequency, $f$ | 1.177 MHz |
| Sigma Antenna Perpendicular Wavelength, $\lambda_\perp$ | 0.105 m |
| Derived Quantity | |
| Alfvén Velocity, $v_A = B/(\mu_0 n_i m_i)$ | $3.38 \times 10^6$ m s$^{-1}$ |
| Ion Thermal Velocity, $v_{ti} = (2T_i/m_i)^{1/2}$ | $1.38 \times 10^4$ m s$^{-1}$ |
| Electron Thermal Velocity, $v_{te} = (2T_e/m_e)^{1/2}$ | $1.19 \times 10^6$ m s$^{-1}$ |
| Ion Cyclotron Frequency, $f_{ci} = eB_0/2\pi m_i$ | 2.59 MHz |
| Electron Cyclotron Frequency, $f_{ce} = eB_0/2\pi m_e$ | 4.76 GHz |
| Electron Plasma Frequency, $\omega_{pe} = (n_e e^2/\epsilon_0 m_e)^{1/2}$ | $6.18 \times 10^{10}$ rad s$^{-1}$ |
| Ion Larmor Radius, $\rho_i = v_{ti}/\omega_{ci}$, | $8.5 \times 10^{-4}$ m |
| Electron Skin Depth, $\delta_e = c/\omega_{pe}$ | $4.85 \times 10^{-3}$ m |
| Thermal Electron Braginskii Collision Frequency, $\nu_{te} = n_e e^4 \ln\Lambda / 2^{5/2}\pi\epsilon_0^2 m_e^{1/2} T_e^{3/2}$ | $4.4 \times 10^6$ s$^{-1}$ |
| Coulomb Logarithm, $\ln\Lambda = \ln(8\pi\epsilon_0^{3/2} T_e^{3/2}/n^{1/2} e^3)$ | 25 |
| Parallel Wavelength, $\lambda_z$ | 2.82 m |
| Dimensionless Quantity | |
| Ion Plasma Beta, $\beta_i = 2\mu_0 n_i T_i / B_0^2$ | $1.67 \times 10^{-5}$ |
| Electron Plasma Beta, $\beta_e = 2\mu_0 n_e T_e / B_0^2$ | $6.69 \times 10^{-5}$ |
| Ion-to-Electron Mass Ratio, $m_i/m_e$ | 1836 |
| Ion-to-Electron Temperature Ratio, $T_i/T_e$ | 0.25 |
| Electron Thermal to Alfvén Velocity Ratio, $v_{te}/v_A$ | 0.35 |
| Perpendicular Wavenumber, $k_\perp \delta_e$ | 0.29 |
| Parallel Wavenumber, $k_z \delta_e$ | $1.1 \times 10^{-2}$ |
| Wave-to-Cyclotron Frequency Ratio, $f/f_{ci}$ | 0.45 |
| Inertial Alfvén Wave Amplitude $E_z/v_A B_0$ | $2.4 \times 10^{-7}$ |

Supplementary Table 1: Experimental parameters, characteristic plasma scales, and dimensionless quantities. Note that Boltzmann's constant is absorbed to give temperatures in units of energy.

Analysis of the linearized cold plasma two-fluid system of equations, in the inertial regime $v_{te}/v_A < 1$, shows the existence of a plane wave solution for inertial Alfvén waves with the dispersion relation [2, 3]

$$\frac{\omega}{k_z} = \pm v_A \frac{\sqrt{1 - \omega^2/\omega_{ci}^2}}{\sqrt{1 + k_\perp^2 \delta_e^2 (1 + i\nu_{te}/\omega)}}, \tag{1}$$

where $\omega_{ci}$ is the ion gyrofrequency, $k_\perp^2 = k_x^2 + k_y^2$ is the squared magnitude of the wave vector perpendicular to $B_0\hat{\mathbf{z}}$, $\delta_e$ is the electron skin depth, and $\nu_{te}$ is the Braginskii collision rate for thermal electrons. Thermal collisions introduce damping. Both finite frequency $\omega/\omega_{ci}$ and finite perpendicular wavenumber $k_\perp \delta_e$ act to decrease the phase speed of inertial Alfvén waves below $v_A$. In the relevant limit $k_z \ll k_\perp$ for our experiment, the group velocity is largely aligned with $\mathbf{B}_0$, so that whatever wave structure exists in the perpendicular $x$-$y$ plane is primarily translated along $\mathbf{B_0}$. However, the perpendicular structure of inertial Alfvén waves is not inconsequential: the dependence of the parallel phase velocity $\omega/k_z$ and parallel group velocity $\partial\omega/\partial k_z$ on $k_\perp$ leads to dispersive behavior. We model the inertial Alfvén waves in this experiment as a superposition of plane waves with different $k_\perp$ values. Following Fourier decomposition, measurements of the Alfvén wave are found to be consistent with the dispersion relation above.

The Sigma antenna we constructed for this experiment excites Alfvén waves by oscillating currents in insulated copper antenna elements that are immersed in the plasma. Antenna elements are connected by low-impedance feeds to external amplifiers. For these experiments, amplifiers drive the antenna at $1.177$ MHz. Using three elements spaced by $2.5$ cm along $\hat{\mathbf{x}}$ allows the antenna to impose structure on the Alfvén wave fields perpendicular to $\mathbf{B_0}$. At the $(x, y)$ location of $g_e(v_z)$ measurements, the Alfvén wave is mostly polarized so that $\mathbf{B}_\perp \approx B_y\hat{\mathbf{y}}$. Consequently, the proceeding overview of the wave analysis will focus primarily on $B_y$ measurements. However, the full analysis uses both $B_y$ and $B_x$ whenever appropriate, e.g. in the calculation of $E_z$.

Electromagnetic fields of the launched wave $\mathbf{B}_\perp$ and $\mathbf{E}_\perp$ perpendicular to $\mathbf{B}_0$ are measured using Elsässer probes [4]. Each probe has B-dot coils to record changes in $\mathbf{B}_x$ and $\mathbf{B}_y$ as well as double probes to record $\mathbf{E}_x$ and $\mathbf{E}_y$. In the analysis presented here, the Elsässer variables [5] are not calculated from the measured wave fields. Instead, $\mathbf{E}_\perp$ and $\mathbf{B}_\perp$ measurements from the two Elsässer probes are analyzed to determine experimentally the phase speed $v_{ph} = \omega/k_z$ of the launched wave modes and compared with predictions from the inertial Alfvén wave dispersion relation in Supplementary Equation (1).

A time slice of the composite $B_y(x, y, z = 1.9\ \mathrm{m}, t)$ measured by Elsässer probe 1, called $B_{y1}$ for brevity, is shown in Supplementary Fig. 1(a). Similarly, we call $B_y(x, y, z = 4.5\ \mathrm{m}, t)$

measured by Elsässer probe 2 $B_{y2}$. The amplitude of $B_{y1}$ is $0.015$ mT. A Fourier transform over $x$ and $y$ gives $\hat{B}_{y1}(k_x, k_y, t)$. The absolute value $|\hat{B}_{y1}|$ of the transformed data from Supplementary Fig. 1(a) is plotted in Supplementary Fig. 1(b). This shows the concentration of wave power is highest in the plane wave modes $(k_x, k_y) = (\pm 50, \pm 19)$ m$^{-1}$. The values for phase velocity, parallel wave vector $k_z$, and parallel wavelength $\lambda_z$ for the plane wave mode $(k_x, k_y) = (50, 19)$ m$^{-1}$ given below are typical for the waves produced at this frequency under these plasma conditions, although it should be noted that we do not approximate that wave as consisting of a single $(k_x, k_y)$ plane wave mode here in the analysis of the experimental measurements. All plane wave modes with measured signal above the noise floor are included whenever appropriate in the analysis of the experimental measurements.

The phase delay for a given mode $(k_x, k_y)$, attributable to the time of flight of the wave between the two Elsässer probes, is found by comparing the time series $\hat{B}_{y1}$ and $\hat{B}_{y2}$. The delay for $(k_x, k_y) = (50, 19)$ m$^{-1}$ is shown in Supplementary Fig. 1(c). Elsässer probe 2, farther from the antenna, records the wave arriving later in time and with diminished amplitude. Phase fronts arriving at probe 2 are delayed by $0.77 \pm 0.03$ $\mu$s. Given the probe spacing of $z_2 - z_1 = 2.56 \pm 0.05$ m, the experimentally determined parallel phase speed for this mode is $v_{ph} = (3.32 \pm 0.14) \times 10^6$ m s$^{-1}$. This experimental value, along with the definition of the parallel phase speed $v_{ph} = \omega / k_z$ and the known $\omega$ driven by the antenna amplifiers, gives an experimental value of $k_z = 2.23 \pm 0.09$ m$^{-1}$ and a parallel wavelength of $\lambda_z = 2.82 \pm 0.11$ m.

To verify that the launched wave mode with $(k_x, k_y) = (50, 19)$ m$^{-1}$ is an inertial Alfvén wave, the experimental values of $v_{ph}$, $k_z$ and $\lambda_z$ are compared with predictions from Supplementary Equation (1) using $v_A = 3.4 \times 10^6$ m s$^{-1}$, $\omega_{ci} = 1.6 \times 10^7$ rad s$^{-1}$, $\delta_e = 5 \times 10^{-3}$ m, and $\nu_{te} = 4.4 \times 10^6$ s$^{-1}$. The predicted values are $v_{ph} = (2.9 \pm 0.3) \times 10^6$ m s$^{-1}$, $k_z = 2.5 \pm 0.3$ m$^{-1}$, and $\lambda_z = 2.5 \pm 0.3$ m. Most of the uncertainty in these values comes from $n_{e0}$. The experimental and predicted values are consistent, supporting the conclusion that the launched wave is an inertial Alfvén wave. A similar analysis can be repeated for each mode $(k_x, k_y)$ with wave power above the noise floor.

Related analysis propagates each plane wave mode $(k_x, k_y)$ in $\hat{B}_{y1}$ to the $z$-location of Elsässer probe 2 using the multiplicative factor $e^{ik_z(z_2 - z_1)}$ and $k_z$ given by Supplementary Equation (1). The result is inverse transformed, and if the wave as a whole is successfully described by a superposition of forward propagating inertial Alfvén plane waves, it should recreate $B_{y2}$. Supplementary Fig. 2 shows a snapshot of the results from this procedure. The measured $B_{y1}$, when propagated to the location of probe E2, successfully reproduces the amplitude, $x$-$y$ structure, and temporal evolution of $B_{y2}$. Notably, sharper features measured in $B_{y1}$, corresponding to higher values of $k_\perp$, are diminished in both the measured and modeled $B_{y2}$. Consistent with

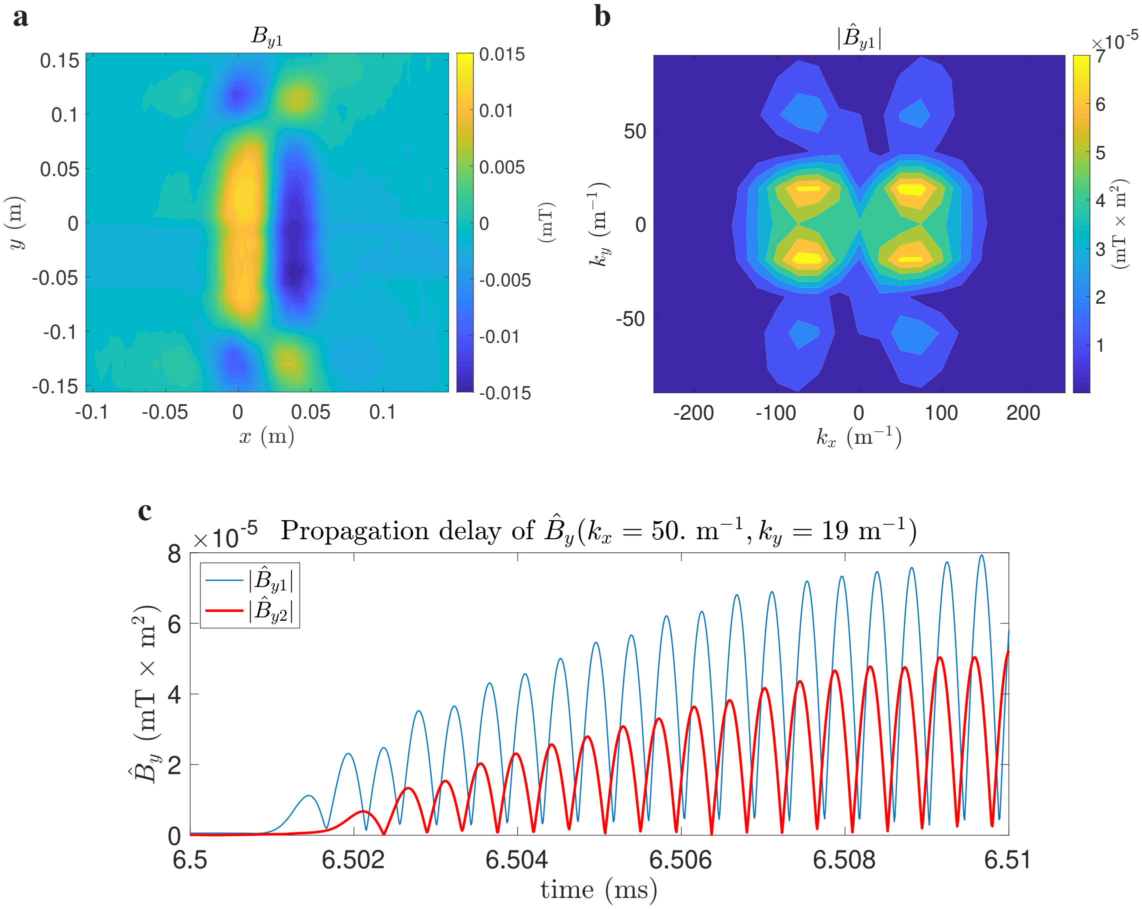


Supplementary Fig. 1: Analysis of Alfvén wave dispersion. **(a)** A time slice of the composite measurement of $B_y$ from Elsässer probe 1. **(b)** A Fourier transform of the same time slice is performed over $x$ and $y$ to reveal modes with the highest concentration of wave power. **(c)** Comparing the time series of $\hat{B}_{y1}$ (blue) and $\hat{B}_{y2}$ (red) for a given $(k_x, k_y)$ shows the time of flight delay of the mode between the two probes.

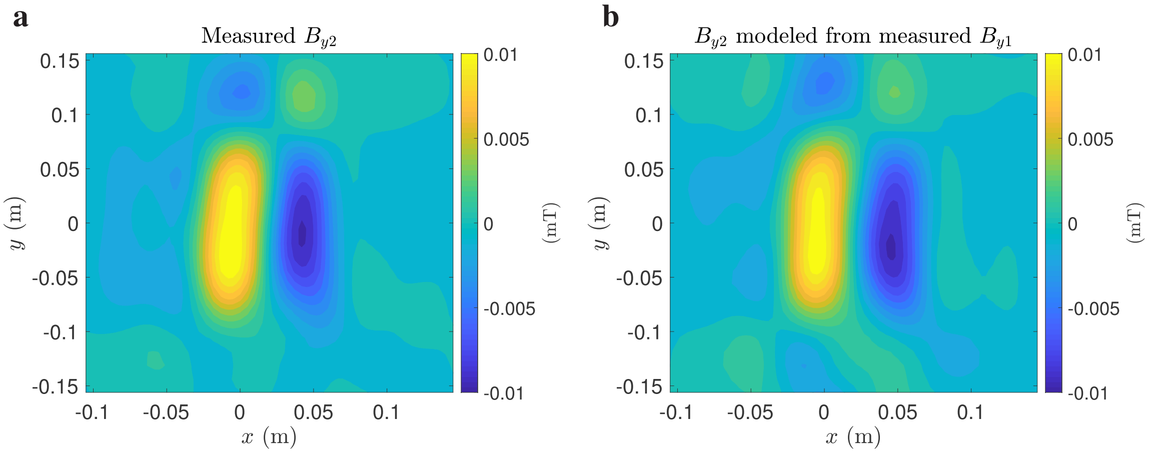


Supplementary Fig. 2: Alfvén wave measurements and model. **(a)** $B_{y2}$ measurements at an instant in time during the Alfvén wave burst. **(b)** A model of $B_{y2}$ at the same instant in time produced from $B_{y1}$ measurements and the inertial Alfvén wave dispersion relation successfully reproduces the measured $B_{y2}$ shown in **(a)**.

this observation, Supplementary Equation (1) predicts higher $k_\perp$ modes are more damped. Success modeling $B_{y2}$ using $B_{y1}$ and Supplementary Equation (1) supports the conclusion that the entire wave pattern measured by Elsässer probes 1 and 2 is a superposition of forward propagating inertial Alfvén waves.

A critical part of the experiment is knowing $E_z$ at the location of $g_e(v_z)$ measurements. $E_z$ is predicted to be small, $E_z/E_x \approx 0.002$. This means an attempt to use a double probe to measure $E_z$ would require an impossible near-perfect alignment of the probe with $\hat{\mathbf{z}}$. Even a $1°$ tilt of the probe toward $\hat{\mathbf{x}}$ would project enough $E_x$ onto the measurement to ruin the effort. Because a direct measurement of $E_z$ is not possible with current measurement capabilities, Ampére's law is used instead to calculate $E_z$ from measurements of $B_x$ and $B_y$. After Fourier transforming in space and time, the $\hat{\mathbf{z}}$ component of Ampére's law gives

$$\hat{E}_z = \frac{c^2(k_y\hat{B}_x - k_x\hat{B}_y)}{\omega K_{zz}} \tag{2}$$

where $c$ is the speed of light, $K_{zz} \approx -\omega_{pe}^2/\omega^2$ is the $(z,z)$ entry of the plasma dielectric tensor, and $\omega_{pe}$ is the electron plasma frequency. An accurate calculation of $E_z$ at the location of $g_e(v_z)$ measurements uses nearby values of $B_x$ and $B_y$. Fortunately, Elsässer probe 2 is less than a meter away in $\hat{\mathbf{z}}$. Success described earlier in this section in recreating $B_{y2}$ using $B_{y1}$ measurements from $2.6\hat{\mathbf{z}}$ m away demonstrates that Elsässer probe 2 data is sufficiently nearby to determine the wave fields at the location of $g_e(v_z)$ measurements.

**Supplementary Methods 3: Measuring the Reduced Electron Velocity Distribution Function.**

The Whistler Wave Absorption Diagnostic (WWAD) provides high-precision measurements of the suprathermal tails of the parallel electron velocity distribution $g_e(v_z)$. It operates by sending a small-amplitude probe wave through a short length of plasma, and the measured damping is used to calculate $g_e(v_z)$. This technique, called wave absorption, can be implemented using any wave absorbed by resonant electrons [6, 7, 8, 9, 10]. In the overdense plasma of the LAPD ($\omega_{pe} > |\omega_{ce}|$), the whistler wave is convenient because it is the only propagating wave mode at frequencies just below $|\omega_{ce}|$ [10]. As a result, wave data at these frequencies can be unambiguously interpreted as whistler waves. The whistler wave is absorbed by Doppler-shifted cyclotron resonant electrons. The resonance condition is

$$v_z k_{\mathrm{w}zr} = \omega_{\mathrm{w}} - |\omega_{ce}|, \tag{3}$$

where $v_z$ is the velocity of resonant electrons, $k_{\mathrm{w}zr}$ is the real component of the whistler wave vector, and $\omega_{\mathrm{w}}$ is the whistler wave frequency. During each $10\ \mu$s measurement of $g_e(v_z)$, $\omega_{\mathrm{w}}$ is chirped to scan $v_z$. Chirping the whistler frequency from $|0.75\omega_{ce}|$ to $|0.95\omega_{ce}|$ scans $v_z$ from $1.8\ v_{te}$ to $14\ v_{te}$, or in energy units, between $5$ eV and $1000$ eV.

The central physical principle of this diagnostic is that damping of the whistler wave is proportional to the number of resonant electrons encountered by the wave. Measured damping of the whistler wave between the transmitting and receiving whistler probes can be used to experimentally determine the reduced electron velocity distribution $g_e(v_z)$ using

$$g_e(v_z) = \frac{2c^2 k_{\mathrm{w}zr}^2}{\pi \omega_{pe}^2 \omega_{\mathrm{w}}} k_{\mathrm{w}zi} = \frac{2c^2 k_{\mathrm{w}zr}^2}{\pi \omega_{pe}^2 \omega_{\mathrm{w}}} \left( \frac{1}{L_{\mathrm{plasma}}} \log \frac{A_T}{A_R} \right), \tag{4}$$

where $\omega_{pe}$ is the electron plasma frequency and $k_{\mathrm{w}zi}$ is the measured damping. The rightmost expression in parentheses shows explicitly the assumption that whistler damping is exponential by relating it to the transmitted and received wave amplitudes, $A_T$ and $A_R$, and the length of plasma $L_{\mathrm{plasma}}$ between the probes. Measured time of flight of the whistler wave between the two probes gives values for $k_{\mathrm{w}zr}$ that agree with the whistler wave dispersion relation [10, 11]. Because every quantity on the right hand side of Supplementary Equation (4) is measured or known, $g_e(v_z)$ can be calculated from experimentally known quantities.

The relative abundance of thermal electrons causes whistler waves with frequencies resonant with thermal velocities to be completely absorbed. As a result, the thermal core of $g_e(v_z)$ is opaque to this diagnostic technique. However, because the resonant acceleration of electrons by inertial Alfvén waves affects suprathermal electrons, the WWAD measures the region of

$g_e(v_z)$ where resonant electron acceleration by inertial Alfvén waves takes place. Although $\omega_{\mathrm{w}}$ reaches frequencies corresponding to $5$ eV, whistler signal does not actually propagate to the receiving antenna until frequencies corresponding to $15$ eV or higher. Measurements of $g_e(v_z > 0)$ are available starting at $15$ eV, while $g_e(v_z < 0)$ is measured at energies above $20$ eV. The asymmetry in the cutoff of measurements occurs because the suprathermal tails of $g_e(v_z)$ are themselves asymmetric. Primary electrons from the cathode cause the $g_e(v_z < 0)$ tail to be greater than $g_e(v_z > 0)$, and so $g_e(v_z < 0)$ becomes opaque at higher energies than $g_e(v_z > 0)$. Discharge current is emitted between the LAPD's cathode and anode seen in Fig. 1(a), so although $g_e(v_z)$ is asymmetric, plasma outside the cathode and anode is current-free.

WWAD measurements in the LAPD of the suprathermal tails of $g_e(v_z)$ are consistent with separate Langmuir probe measurements and kinetic theory. Prior measurements of $g_e(v_z)$ were used to find values of $n_e$ and $T_e$ that agree with simultaneous Langmuir probe measurements [10]. Additionally, WWAD measurements of $g_e(v_z)$ resolved in Alfvén wave phase isolated perturbations to $g_e(v_z)$ caused by the Alfvén wave that are well-described by linear kinetic theory [12].

**Supplementary Methods 4: Applying the Field-Particle Correlation Technique to Experimental Measurements.**

The field-particle correlation technique is an innovative analysis method that uses co-located (in configuration space) electromagnetic field and particle velocity distribution function measurements to determine the rate of energy transfer between the electromagnetic fields and particles [13, 14, 15]. The Maxwell-Boltzmann equations govern the dynamics and evolution of a kinetic plasma. Under the weakly collisional conditions of the auroral magnetosphere, we can drop the collision term in the Boltzmann equation, which is unnecessary to describe the collisionless transfer of energy between fields and the accelerated suprathermal electrons, to obtain the Vlasov equation. Because the linear electron cyclotron frequency $f_{ce} = 4760$ MHz in the experiment is more than three orders of magnitude greater than the Alfvén wave driving frequency $f = 1.177$ MHz, we can average the Vlasov equation over the electron gyrophase to obtain the gyroaveraged Vlasov equation. Subsequently, we integrate over the perpendicular component of velocity to obtain the evolution equation for the reduced parallel velocity distribution $g_e(x, y, z, v_z, t)$ [11],

$$\frac{\partial g_e}{\partial t} + v_z \frac{\partial g_e}{\partial z} - \frac{e}{m_e} E_z \frac{\partial g_e}{\partial v_z} = 0. \tag{5}$$

Note the Supplementary Equation (5) is formally dependent on the guiding center coordinates $(X, Y)$ in the plane perpendicular to the strong axial magnetic field, but because the electron gyroradius, $\rho_e = v_{te}/\omega_{ce} = 0.004$ cm, is much less than the accuracy of our spatial position measurements, we can approximate the guiding center position as the spatial position in the perpendicular plane, $(X, Y) \simeq (x, y)$.

To diagnose the energy transfer between fields and electrons in this experiment, we define the reduced parallel phase-space energy density, $w_e(x, y, z, v_z) \equiv m_e v_z^2 g_e(x, y, z, v_z)/2$, for the electrons. Note that the standard form of the field-particle correlation technique is formulated using the full electron phase-space energy density, $m_e v^2 f_e/2$, defined using the full distribution function $f_e(\mathbf{v})$, rather than the reduced distribution function $g_e(v_z)$. The velocity-space signature of the net resonant energy transfer caused by $E_z$ is the same for both cases, however, because the $v_x^2$ and $v_y^2$ contributions to the field-particle correlation sum to zero when integrated over $v_z$ [14]. Therefore, we can apply our intuition from the previous literature on field-particle correlations to the reduced parallel case here, where our experimental measurements do no separately resolve $v_x$ or $v_y$. Multiplying the reduced parallel Vlasov equation by $m_e v_z^2/2$ yields an equation for the evolution of the reduced parallel phase-space energy density $w_e(v_z)$,

$$\frac{\partial w_e}{\partial t} = -\frac{m_e v_z^3}{2} \frac{\partial g_e}{\partial z} + \frac{e v_z^2}{2} \frac{\partial g_e}{\partial v_z} E_z, \tag{6}$$

dependent on the parallel electric field $E_z$ and the reduced parallel distribution function $g_e(v_z)$.

To determine the dominant contribution to the rate of change of $w_e(v_z) = w_e(x_0, y_0, z_0, v_z)$ averaged over the Alfvén wave period $T$ at the single point of measurement $(x_0, y_0, z_0)$, we can Fourier transform the distribution function in time over the Alfvén wave period. The discrete Fourier transform of the distribution function over $T$ for $g_e(v_z, t_j)$ sampled at $t_j = j\Delta t$ for $j = 0, 1, \dots, N-1$ and $\Delta t = T/N$ is given by

$$\hat{g}_e(v_z, \omega_n) = \sum_{j=0}^{N-1} g_e(v_z, t_j) e^{i\omega_n t_j} \tag{7}$$

where $\omega_n = n\omega$, $n = -N/2, \dots, N/2$, and the Alfvén wave frequency is $\omega = 2\pi/T$. The time evolution of each Fourier harmonic is given by

$$g_{en}(v_z, t_j) = \left[ \hat{g}_e(v_z, \omega_n) e^{-i\omega_n t_j} + \hat{g}_e^*(v_z, \omega_n) e^{i\omega_n t_j} \right] /2. \tag{8}$$

The first few Fourier harmonics from the measured $g_e(v_z, t)$ are well ordered, such that $g_{e0}(v_z, t_j) \gg g_{e1}(v_z, t_j)$ and $g_{e1}(v_z, t_j) \gg g_{en}(v_z, t_j)$ for $n \geq 2$.

Expanding the electron distribution function into its Fourier harmonics $g_e(v_z, t) = g_{e0}(v_z, t_j) + g_{e1}(v_z, t_j) + \cdots$, substituting into Supplementary Equation (6), and keeping only the lowest order nonlinear term, we obtain an equation for the evolution of the reduced parallel phase-space energy density $w_e(v_z)$,

$$\frac{\partial w_e}{\partial t} = -\frac{m_e v_z^3}{2}\frac{\partial g_{e1}}{\partial z} + \frac{e v_z^2}{2}\frac{\partial g_{e0}}{\partial v_z}E_z + \frac{e v_z^2}{2}\frac{\partial g_{e1}}{\partial v_z}E_z. \tag{9}$$

At the point of measurement $(x_0, y_0, z_0)$, this equation tells us that the rate of change of parallel phase-space energy density dominantly depends on the parallel electric field $E_z$ along with the constant $g_{e0}(v_z)$ and fundamental mode $g_{e1}(v_z, t)$ contributions to the Fourier expansion.

Since $E_z(t)$ and $g_{e1}(v_z, t)$ both oscillate at the Alfvén wave frequency $\omega$, when this equation is averaged over the Alfvén wave period $T$, the first two terms on the right-hand side of Supplementary Equation (9) contribute nothing to the net electron energization rate. The third term, a product of two quantities oscillating at the same frequency $\omega$, results in oscillations at frequency $2\omega$ as well as a constant term. This constant term, the amplitude of which depends on the relative phases of $E_z(t)$ and $g_{e1}(v_z, t)$, is the dominant contribution to the time-averaged rate of energy transfer between electrons and the parallel electric field $E_z$ of the Alfvén wave. Therefore, to isolate the dominant contribution to the net energy transfer rate between the Alfvén wave and the electrons, the correlation

$$C_{E_z}(v_z) = Corr\left(\frac{e v_z^2}{2}\frac{\partial g_{e1}(v_z, t)}{\partial v_z}, E_z(t)\right), \tag{10}$$

is computed, consisting of an unnormalized time average over the full Alfvén wave period. Note that the integration of the reduced parallel correlation over parallel velocity yields the rate of work done by the parallel electric field on the electrons, $j_{ez}E_z = \int dv_z C_{E_z}(v_z)$.

The resulting correlation $C_{E_z}(v_z)$ yields the *velocity-space signature* of energy transfer to the electrons as a function of the parallel velocity $v_z$. The correlation is positive in regions of velocity space where the parallel phase-space energy density increases due to work on the electrons by the parallel electric field, and is negative where the parallel phase-space energy density decreases. Previous numerical [15] and observational [16] applications of the field-particle correlation technique to weakly collisional heliospheric plasma turbulence has demonstrated that energization of particles by the Landau resonance leads to a characteristic bi-polar velocity-space signature, with a decrease in $w_e(v_z)$ at velocities just below the resonant parallel phase velocity, and an increase just above the resonant velocity. This is the characteristic signature of electron energization that we are seeking in our laboratory experiments of auroral electron

acceleration by Alfvén waves.

**Supplementary Methods 5: Gyrokinetic Simulations of Electron Acceleration by Inertial Alfvén Waves.**

The gyrokinetic simulation code `AstroGK` [17] is used to evolve the electron velocity distribution function self-consistently as an inertial Alfvén wave propagates through the periodic simulation domain. Plasma parameters and wave properties are chosen to match the LAPD experiments, with $\beta_i = 1.67 \times 10^{-5}$ and $T_i/T_e = 0.25$ and perpendicular wavenumber of the wave $k_\perp \rho_i = 0.05$ with $k_z/k_\perp \ll 1$. Linear gyrokinetic theory predicts a normalized complex wave frequency of $\overline{\omega}_{\mathbf{GK}} \equiv \omega/k_z v_A = (0.9708, -7.195 \times 10^{-4})$, leading to a very weak normalized damping rate of $\gamma/\omega = 7.41 \times 10^{-4}$. We specify collisional coefficients for the ions and electrons in the collision operator [18, 19] in terms of the wave frequency, as $\nu_i/\omega = \nu_e/\omega = 4.2 \times 10^{-3}$, yielding a regime of weak collisionality. The numerical resolution of this 3D-2V gyrokinetic simulation is $(n_x, n_y, n_z, n_\lambda, n_\varepsilon, n_s) = (10, 10, 256, 64, 32, 2)$, where velocity space coordinates are $\lambda = v_\perp^2/v^2$ and $\varepsilon = v^2/2$, uniform Maxwellian equilibria are chosen for ions and electrons, and a realistic mass ratio is specified, $m_i/m_e = 1836$. The domain is a periodic box of size $L_\perp^2 \times L_z$, elongated along the straight, uniform mean magnetic field $\mathbf{B}_0 = B_0\hat{\mathbf{z}}$, where all quantities may be rescaled to any parallel dimension satisfying $L_z/L_\perp \gg 1$, and with $L_\perp = 40\pi\rho_i$.

A plane, inertial Alfvén wave with wavevector $(k_x/k_{\perp 0}, k_y/k_{\perp 0}, k_z/k_{z0}) = (1, 0, 8)$ is initialized with the exact kinetic eigenfunction from linear gyrokinetic theory [20]. To eliminate any transient behavior arising from the initialization that does not agree with the properties of the inertial Alfvén wave mode, we evolved the simulation linearly for five periods with enhanced collision frequencies $\nu_i = \nu_e = 4.2 \times 10^{-2} k_z v_A$, as successfully used in previous studies using eigenfunction initialization of wave modes [21, 22].

After the elimination of the transient, the simulation is restarted nonlinearly with the lower collisionality $\nu_s/\omega = 4.2 \times 10^{-3}$. At a single point in the simulation domain, the perturbed complementary gyrokinetic electron distribution function $g_e$ [17] and the parallel electric field $E_z$ are output at uniform time intervals. The parallel field-particle correlation on the gyrotropic velocity space $C_{E_z}(v_z, v_\perp)$ is computed over the three wave periods $10 \leq t/T \leq 13$ in the simulation, and the result is plotted in Figure 3(b). Integration over the perpendicular velocity coordinate yields the reduced parallel field-particle correlation $C_{E_z}(v_z)$, a quantity that can be compared directly to the results of the electron acceleration experiments on the LAPD.

Note that, because of the periodic domain, the gyrokinetic simulation results are relevant to measurements made at an asymptotically large distance $z/\lambda \gg 1$ from source of the waves.

This condition does not hold true for the experimental measurements, which were measured at a distance $z/\lambda \simeq 2$. Furthermore, gyrokinetic theory is valid in the limit $\omega/\omega_{ci} \ll 1$, so the decrease in the real frequency due to the effects of the cyclotron resonance as $\omega/\omega_{ci} \to 1$, as clear from Supplementary Equation (1), are not included in the gyrokinetic simulation. The resonant parallel velocity in the gyrokinetic simulation is given by $\omega_{\text{GK}}/k_z v_{te} = 2.71$, as evident in Fig. 3(b) and (c). Using the linear Vlasov-Maxwell dispersion relation to account for the finite frequency value $\omega/\omega_{ci} = 0.45$ from the experiment, a complex frequency value of $\overline{\omega}_{\text{VM}} = (0.863, -1.50 \times 10^{-3})$ is obtained. The finite frequency effects lead to a reduction in the parallel phase velocity, and thereby a reduction in the resonant parallel electron velocity to $\omega_{\text{VM}}/k_z v_{te} = 2.4$. The modeling of the electron velocity distribution using Liouville mapping, as described in Supplementary Methods 7, uses this more physically accurate value of $\overline{\omega}_{\text{VM}}$ in prescribing the inertial Alfvén wave.

**Supplementary Methods 6: Laplace Transform Solution of Field-Particle Correlation at Finite Distance.**

Assume an inertial Alfvén wave exists with an electric field parallel to $\mathbf{B}_0$ with the form $E_z = E_{z0} e^{i\mathbf{k}\cdot\mathbf{x} - i\omega t}$, where $\mathbf{k}$ is complex, $\omega$ is real, and both satisfy the inertial Alfvén wave dispersion relation. $E_z$ is the only wave field that explicitly enters the reduced gyroaveraged equation for the evolution of $g_e$ in Supplementary Equation (5). The distribution $g_e(x, y, z, v_z, t)$ is separated into a uniform static background $g_{e0}(v_z)$ and a small perturbation caused by the inertial Alfvén wave the varies in space and time $g_{e1}(x, y, z, v_z, t)$. The linearized form of Supplementary Equation (5) is

$$\frac{\partial g_{e1}}{\partial t} + v_z \frac{\partial g_{e1}}{\partial z} - \frac{e}{m_e} E_z \frac{\partial g_{e0}}{\partial v_z} = 0. \tag{11}$$

To account for the finite interaction length of electrons with the Alfvén waves in this experiment, Supplementary Equation (11) is solved using a Laplace transform over $z$. Because the duration of the inertial Alfvén wave burst is much longer than the lifetime of electrons traversing the LAPD, a Fourier transform is applied over time. The result of applying these transforms to Supplementary Equation (11) is

$$-i\omega \tilde{g}_{e1} + v_z p \tilde{g}_{e1} - v_z \int_{-\infty}^{\infty} dt e^{i\omega t} g_{e1}(x, y, z = 0, v_z, t) - \frac{e}{m_e} \tilde{E}_z \frac{\partial g_{e0}}{\partial v_z} = 0, \tag{12}$$

where a tilde is used to denote the Laplace and Fourier transformed versions of the quantities $g_{e1}$ and $E_z$, and $p$ is the Laplace transform conjugate variable of $z$. The boundary condition at

the antenna $g_{e1}(x,y,z=0,v_z,t)$ is introduced in Supplementary Equation (12) by the Laplace transform and acted on by the Fourier transform.

Supplementary Equation (12) can be solved algebraically for $\tilde{g}_{e1}$, and the real space solution $g_{e1}$ is produced by carrying out inverse Laplace and Fourier transforms. The result is

$$\begin{aligned} g_{e1}(x,y,z,v_z,t) =& \frac{1}{2\pi}\int_{-\infty}^{\infty} d\omega e^{-i\omega t} e^{i\omega z/v_z} \int_{-\infty}^{\infty} dt e^{i\omega t} g_{e1}(x,y,z=0,v_z,t) \\ &+ \frac{ie}{m_e}\frac{\partial g_{e0}}{\partial v_z} E_z \left(\frac{1-e^{i(\omega/v_z - k_z)z}}{\omega - k_z v_z}\right). \end{aligned} \tag{13}$$

The first line on the right hand side of Supplementary Equation (13) is the boundary condition propagated ballistically to $(x,y,z)$. The second line is the linear wave-particle interaction. As expected, the numerator of the wave-particle interaction is zero when the interaction length $z=0$. The resonant denominator allows the wave particle interaction to grow for electrons at or near the inertial Alfvén wave phase velocity $v_z = \omega/k_z$.

The solution for $g_{e1}$ discussed so far only applies to electrons originating at the Alfvén wave antenna and traveling from there to the location of $g_e$ measurements. In other words, the solution described above is valid for $g_{e1}(v_z > 0)$. Following a similar procedure, a solution is produced for $g_{e1}(v_z < 0)$.

To evaluate Supplementary Equation (13), a boundary condition $g_{e1}(x,y,z=0,v_z,t)$ is needed. While measurements of the perturbation to the distribution function are not performed at the antenna, a reasonable approximation can be generated by requiring that the boundary condition be consistent with the fluid properties of the inertial Alfvén wave. Specifically, $g_{e1}(x,y,z=0,v_z,t)$ must include the parallel current of the Alfvén wave required by two fluid theory $J_z = in_e e^2 E_z/(m_e\omega)$ [23]. To this end, the boundary condition is given the form

$$g_{e1}(x,y,z=0,v_z,t) = \frac{ie}{m_e\omega} E_z(x,y,z=0,t)\frac{\partial g_{e0}}{\partial v_z}. \tag{14}$$

The first moment of this term reproduces the required $J_z$ of the inertial Alfvén wave.

To this point, collisions have been omitted from the discussion of $g_{e1}$. Collisions are introduced using a velocity-dependent Krook collision operator [2, 11]. This operator adds a collision term to the right hand side of Supplementary Equation (11) with the form $-\nu(v_z)g_{e1}$ where $\nu(v_z)$ is the velocity-dependent Coulomb collision rate. The result of the collision term is $\omega \rightarrow \omega + i\nu(v_z)$ in Supplementary Equation (13) and Supplementary Equation (14), except

in the Fourier kernals $e^{i\omega t}$ and $e^{-i\omega t}$ and the implicit $\omega$ in $E_z$. By adding collisions, this generalizes our solution to the collisionless Vlasov equation to be a solution to the weakly collisional Boltzmann equation.

Alfvén wave antennas are often inefficient because Alfvén wavelengths are usually much longer than the antennas that can be installed through typical vacuum ports. This experiment is no exception. As a result, some of the power injected into the plasma by the antenna is likely not deposited into the inertial Alfvén wave mode, and some portion of the measured $g_{e1}$ will be due to non-Alfvénic oscillations. In earlier work [11], non-Alfvénic perturbations were modeled using the velocity perturbation of electrons traversing a voltage drop across the antenna. This effect is included as a homogeneous solution to Supplementary Equation (11) in the particular solution for $g_{e1}$. Because the amplitude and phase of the voltage drop across the antenna are not measured, they are allowed to be adjusted to minimize the $\chi^2$ difference between the measured and modeled $g_{e1}$. The amplitude and phase of the voltage drop across the Alfvén wave antenna are the only free parameters in the entire model for $g_{e1}$. The voltage drop across the antenna is typically $1$ volt or less. Of all the contributions to the modeled $g_{e1}$, the largest is the wave-particle interaction in Supplementary Equation (13). The wave-particle interaction requires a finite interaction length to develop, which ultimately explains why the resonant signature in $C_{E_z}$ evolves near the antenna and the bipolar signature of resonant electron acceleration asymptotically approaches the resonant velocity as distance from the antenna increases.

The solution for $g_{e1}$ in Supplementary Equation (13) is for a single inertial Alfvén plane wave mode. As discussed in Supplementary Methods 2, the inertial Alfvén waves generated in this experiment are a superposition of multiple plane waves. Because of this, a realistic model requires all the $g_{e1}$'s, each attributable to a separate inertial Alfvén plane wave mode, to be summed. The result is used for comparing with the measured $g_{e1}$ as well as for predictions of $C_{Ez}$ in this experiment.

**Supplementary Methods 7: Liouville Mapping of the Electron Velocity Distribution.**

We can model the effect of the inertial Alfvén wave on the electron velocity distribution by a mapping of phase space through the wave fields according to Liouville's theorem, a technique we denote here as Liouville mapping [24, 25]. In this technique, we build up each point in the electron velocity distribution at the spatial point $z_{obs} > 0$ and time $t_{obs} > 0$ of observation by mapping backwards in time according to the Vlasov equation (where the trajectory of a small volume in 3D-3V phase space at velocity $\mathbf{v}_i$ is determined by its velocity and the Lorentz force law). This backwards time integration is continued until the small volume of phase space reaches the time $t = 0$ at which the wave was initially launched at $z = 0$ from the antenna, or until the particle reaches the antenna at $z = 0$. At the end of this time integration, we obtain the velocity of that point in phase space, $\mathbf{v}_f$. In the absence of collisions, the relevant case for the

Vlasov equation, we can invoke Liouville's theorem to set the phase space density at $\mathbf{v}_i$ at the observation time and position equal to the phase space density at $\mathbf{v}_f$ of the "initial distribution" at the end of the backwards time integration.

Therefore, one simply needs to determine the form of the "initial distribution" from physical considerations. We take our initial conditions for the electrons at $t = 0$ to be an isotropic Maxwellian distribution with $T_e = 4$ eV everywhere. We consider that the antenna turns on at $t = 0$, launching an inertial Alfvén wave that propagates from $z = 0$ in the positive $z$ direction with the appropriate parallel phase velocity given by the Vlasov-Maxwell dispersion relation, $\overline{\omega}_{\text{VM}} = \omega/k_z v_A = 0.863$. See discussion at the end of Supplementary Methods 5 about the difference between the complex linear inertial Alfvén wave frequencies using the linear gyrokinetic dispersion relation [20] or the linear Vlasov-Maxwell dispersion relation [26]. For the Liouville mapping results here, we choose on the Vlasov-Maxwell approach. For each volume of phase space (at velocity $\mathbf{v}_i$ at the start of the backwards integration) that remains at $z > 0$ for all $t \geq 0$ as it is mapped back in time, we simply need to integrate back in time to $t = 0$. In this case, the phase-space density at the position and time of observation at velocity $\mathbf{v}_i$ is simply set equal to the phase-space density of the initial Maxwellian equilibrium at velocity $\mathbf{v}_f$, or $f_{obs}(\mathbf{v}_i, t_{obs}) = f_0(\mathbf{v}_f, 0)$.

On the other hand, for volumes of phase space (at velocity $\mathbf{v}_i$ at the start of the backwards integration) that reach the antenna at $z \leq 0$ before reaching $t = 0$ when mapped back in time, we require a more sophisticated treatment of the "initial distribution". We take the antenna to be a physical barrier at $z < 0$, so that we can only sample the electron distribution at $z \geq 0$. Therefore, we integrate the electron phase space trajectories backwards in time only until that phase-space volume reaches $z = 0$. We adopt an idealized approximation that the wave appears at $z = 0$ and can only effect the electron distribution after integration over a finite distance along $z > 0$. Following the full solution of the linearized kinetic equation for an inertial Alfvén wave generated by an antenna outlined in Schroeder *et. al* (2017) [11], we account for two specific "near-antenna" effects that can influence the electron velocity distribution measured at the probe: (i) a "fluid" component to the boundary condition that arises from the varying electrostatic potential $\phi(t)$ of the Alfvén wave driven by the antenna; and (ii) a "homogeneous" term in the solution that yields a boundary condition describing the non-Alfvénic response of the plasma in the sheath region at the antenna surface.

The fluid contribution to the $z = 0$ boundary condition yields a standard Boltzmann correction to the Maxwellian distribution due to the electrostatic potential $\phi(t)$ of the Alfvén wave at the time $t_{z=0}$ when the phase-space volume reaches the antenna. The electrostatic potential at the antenna is determined by integrating from beyond the leading edge of the wave at $z_*$ back to $z = 0$, $\phi(z = 0, t_{z=0}) = -\int_{z_*}^{0} E_z(z, t_{z=0}) dz$. Because the wave launched from the antenna

at $z = 0$ has no length of interaction over which it can modify the electron velocity distribution, the only effect on the distribution at $z = 0$ is the varying electrostatic potential needed to launch the wave. Therefore, in this case, the phase-space density at the position and time of observation at velocity $\mathbf{v}_i$ is simply set equal to the phase-space density of the Maxwellian equilibrium at velocity $\mathbf{v}_f$ modified by the electrostatic potential,

$$f_{obs}(\mathbf{v}_i, t_{obs}) = f_{z=0}(\mathbf{v}_f, t_{z=0}) = \frac{n_0}{\pi^{3/2} v_{te}^3} e^{-[\mathbf{v}_f^2/v_{te}^2 - e\phi(t)/T_e]}. \tag{15}$$

The homogeneous boundary conditions adds a perturbation $\delta f_h$ to $f_{obs}(\mathbf{v}_i, t_{obs})$ due to the varying potential change through the plasma sheath given by

$$\delta f_h(\mathbf{v}_i, t_{obs}) = \frac{eV_{s0}}{m_e v_z} \frac{\partial f_0(\mathbf{v}_f, t_{z=0})}{\partial v_z} e^{i\omega(z_{obs}/v_z - t_{obs}) + i\alpha_s} \tag{16}$$

where $V_{s0}$ is the amplitude of the plasma sheath potential and $\alpha_s$ is the phase of the sheath potential relative to that of the Alfvén wave [11]. The exponent gives the phase of the sheath potential ballistically propagated to the position $z_{obs}$ and time $t_{obs}$ at which the electron velocity distribution is observed. The model shown in Fig. 3(f)–(h) uses $V_{s0} = 0.28$ V and $\alpha_s = -\pi/2$.

Because the resonant acceleration of the electrons is governed by the parallel component of the electric field $E_z$, we do not model the effect of the perpendicular electric field on the electron distribution (which would lead to a conservative oscillation of the distribution in the perpendicular direction that averages out over one period). Rather, we model only the parallel electric field at $z \geq 0$ by

$$E_z(z,t) = \begin{cases} E_{z0} \sin(k_z z - \omega t), & z \leq 0 \leq \omega t/k_z \\ 0, & z \geq \omega t/k_z \end{cases} \tag{17}$$

The Liouville mapping code was validated by comparison to published results in Kletzing (1994) [24]. The dimensionless parameters of the Liouville mapping calculation are $\beta_i = 1.67 \times 10^{-5}$, $m_i/m_e = 1836$, $v_{te}/v_A = 0.35$, perpendicular wavenumber $k_x \delta_e = 0.29$, parallel wavenumber $k_z \delta_e = 9.63 \times 10^{-3}$, wave frequency $\omega/k_z v_A = 0.863$, and Alfvén wave amplitude $E_z' = cE_z/v_A B_0 = 2.4 \times 10^{-7}$. In Fig. 3(f)–(h), the electron velocity distribution function $f_e$ and $E_z$ needed to compute field-particle correlation $C_{E_z}(v_\perp, v_z)$ are determined at $z_{obs}/\delta_e = 1386$, or $z_{obs}/\lambda_z = 2.12$, with the correlation taken over 100 uniformly spaced samples in time over the time range $2.13 \leq t/T \leq 3.13$, where $T$ is the period of the Alfvén wave. The electron velocity distributions plotted in Fig. 4 are taken (a) at $z_{obs}/\delta_e = 1386$ (equivalently $z_{obs}/\lambda_z = 2.12$ or $z_{obs} = 5.27$ m), and $t_{obs}/T = 3.13$ and (b) at $z_{obs}/\delta_e = 6930$

(equivalently $z_{obs}/\lambda_z = 10.61$ or $z_{obs} = 26.35$ m) and $t_{obs}/T = 11.63$. The nonlinear trapping width [27] in parallel velocity, shown by the dashed vertical gray lines in Fig. 4, is given by $\Delta v_z/v_{te} = \pm\sqrt{e\phi/T_e} = \pm 0.19$, showing good agreement with the extent in $v_z$ of the effect of the wave on the electron velocity distribution.

**Supplementary Methods 8: Comments on Connection Between the Experiment and the Three Modeling Approaches.**

A few brief notes on the connection between the LAPD lab experiment and the `AstroGK` simulation, Laplace transform solution using analytical theory, and Liouville mapping models are worthwhile mentioning here. First, the gyrokinetic simulation self-consistently determines the electromagnetic fields from the electron (and ion) velocity distributions, but it does not include the finite frequency effects (due to finite value of $k_z\rho_i$, which results in a finite value of $\omega/\omega_{ci}$), which reduce the wave frequency, in this case to 89% of the gyrokinetic wave frequency (see discussion at the end of Supplementary Methods 5). Furthermore, the gyrokinetic simulation results are also effectively in the limit of an asymptotically large distance from the antenna, so they do not account for a finite distance from the antenna. Second, the Laplace transform solution directly takes into account the finite distance from the antenna, showing that the zero crossing of $C_{E_z}(v_z)$ shifts away from the resonant velocity towards zero. Finally, the Liouville mapping result can be used, assuming an inertial Alfvén wave frequency that includes the finite-frequency effects (and a similarly altered value of $k_z$ for the given experimental value of the driving wave frequency) to make a direct comparison to the experimental results. Although the dimensional amplitude of the reduced parallel correlation $C_{E_z}(v_z)$ from the Liouville mapping result is about a factor of two smaller than the experimental determination, this is likely due to the fact that the Liouville modeling used a single-temperature Maxwellian equilibrium velocity distribution and only included the dominant Alfvén wave mode. The experimental measurements found a more complicated equilibrium velocity distribution and the analysis included additional subdominant wave modes generated by the antenna, so this likely is the root of the overall amplitude difference. But, the most important aspect of the comparison between the Liouville mapping results and the experiment is that a simple Maxwellian equilibrium model quantitatively recovers the velocity of the zero crossing and peak of $C_{E_z}(v_z)$, the key features that confirm the characteristic velocity-space signature of Landau-resonant electron energization. An additional benefit of the Liouville mapping modeling is that we can trivially extend the calculation to determine the perturbed electron distribution function, specifically the generation of a population of accelerated electrons, at distances beyond the physical length of the experimental chamber, as shown in Fig. 4.

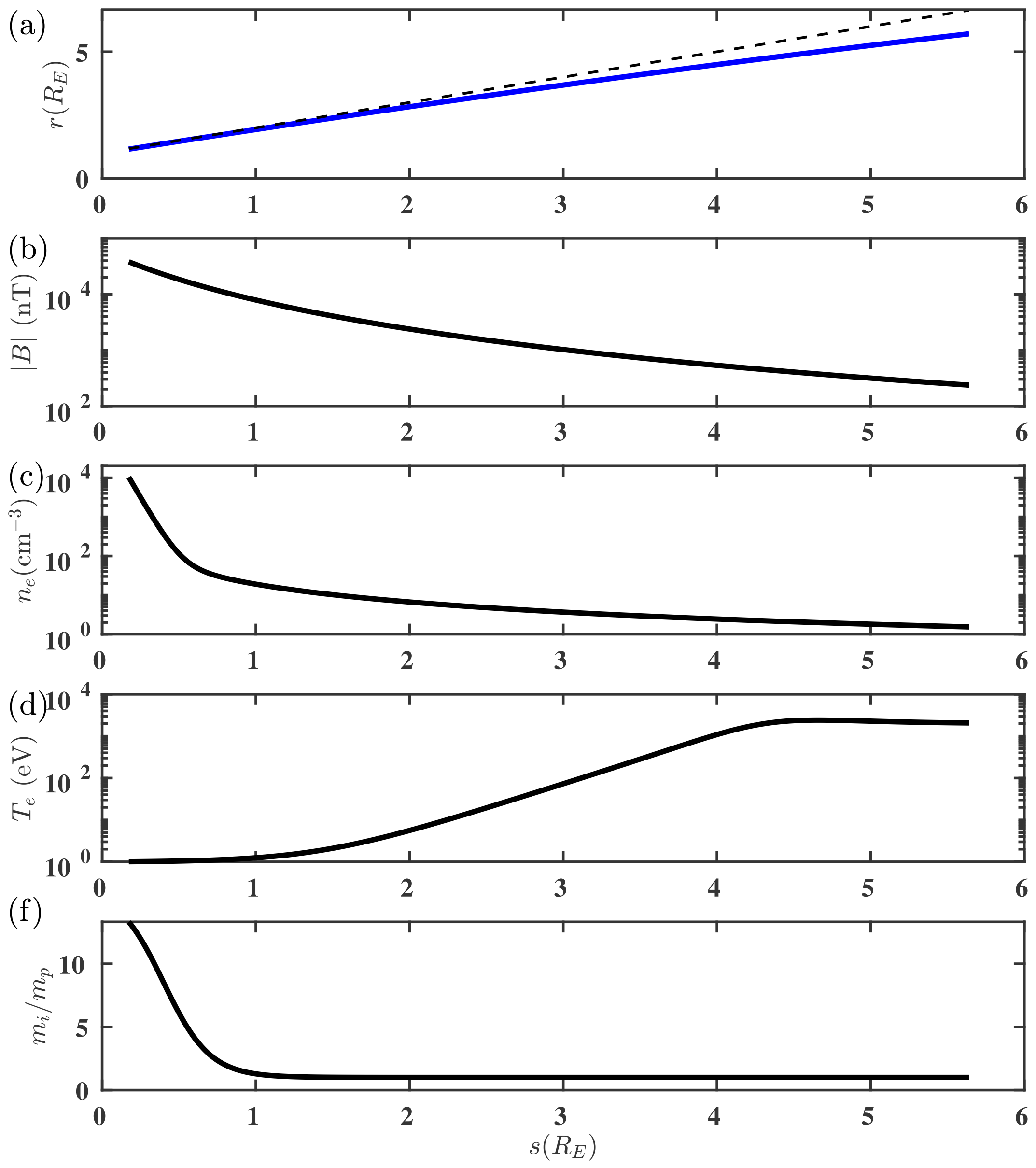


Supplementary Fig. 3: Auroral zone plasma parameters. Model plasma parameters vs. distance $s$ along the $L = 8.5$ field line above the Earth's surface, showing (a) geocentric radius $r$ (blue) with $r = s$ (dotted) plotted for comparison, (b) magnetic field magnitude $|B|$, (c) electron density $n_e$, (e) electron temperature $T_e$, and (f) the normalized effective ion mass $m_i/m_p$.

**Supplementary Methods 9: Model of Auroral Acceleration Region.**

To make a meaningful quantitative comparison between the rate of energization of electrons in the experiment to that in the auroral acceleration region [28, 29] of the Earth's magnetosphere, we present here a model of the plasma parameters in the Earth's magnetosphere. We take the Earth's magnetic field within the inner magnetosphere $r \lesssim 6\ \mathrm{R_E}$ to be a standard dipole magnetic field with a dipole moment $3.12 \times 10^{-5}$ T $\mathrm{R_E}^3$ and no axis tilt. We use the empirically determined electron density profile from Kletzing, Mozer, and Torbert (1998)[1], given by

$$n_e(z) = n_0 e^{-(z-z_0)/h} + n_1 z^{-1.55} \tag{18}$$

where $n_0 = 6 \times 10^4$ cm$^{-3}$, $n_1 = 1.34 \times 10^7$ cm$^{-3}$, $z_0 = 318$ km, and $h = 383$ km. The electron temperature profile is a version of the profile from Kletzing and Torbert (1994) [30] modified to asymptote to plasma sheet temperatures in the keV range, given by

$$T_l(z) = T_1 e^{z/h_0} + T_0 \tag{19}$$

$$T_e(z) = T_l w + T_{ps}(1 - w) \tag{20}$$

where the parameters $T_0 = 1$ eV, $T_1 = 0.0135$ eV, and $h_0 = 2000$ km are chosen such that the electron temperature is 1 eV at $z = 300$ km and 3 eV at $z = 10000$ km, and the weighting

$$w = \frac{1}{2}\left[1 - \tanh\left(\frac{z - z_{ps}}{\Delta z}\right)\right] \tag{21}$$

yields a smooth transition from the lower altitude temperature variation given by Kletzing and Torbert (1994) [30] to a constant plasma sheet temperature of $T_{ps} = 2$ keV over a transition length $\Delta z = 0.3\ \mathrm{R_E}$ centered at $z_{ps} = 3.75\ \mathrm{R_E}$. We make the simplifying assumption that $T_i(z) = T_e(z)$, but we note that the electron physics in the inertial regime is generally insensitive to the value of $T_i$. Finally, we employ a model of the ion composition inspired by Chaston (2006) [31] that transitions from $O^+$ as the dominant ion species in the ionosphere to $H^+$ at higher altitudes, given by

$$\frac{n_O}{n_i} = \frac{1}{2}\left[1 - \tanh\left(\frac{z - z_i}{h_i}\right)\right] \tag{22}$$

where $z_i = 2370$ km and $h_i = 1800$ km. The effective ion mass $m_i$ as a function of altitude is given by $m_i = (n_H m_p + n_O m_O)/n_i$, where the total ion density is approximated by $n_i = n_H + n_O$. From this model, the resulting profiles of geocentric radius $r$, magnetic field magnitude $|B|$, electron density $n_e$, electron temperature $T_e$, and the normalized effective ion mass $m_i$ vs. distance along the $L = 8.5$ field line $s$ are plotted in Supplementary Fig. 3.

| Experimental and auroral acceleration region parameters | | |
|---|---|---|
| Quantity | Experiment at $z/\lambda \simeq 2$ | Auroral Acceleration Region, $z = 2.56R_E$ |
| Background plasma parameters | | |
| Magnetic Field, $B_0$ | 0.17 T | 1140 nT |
| Electron Density, $n_{e0}$ | $1.2 \times 10^{18}$ m$^{-3}$ | $4 \times 10^6$ m$^{-3}$ |
| Electron Temperature, $T_e$ | $4 \pm 1$ eV | 50 eV |
| Ion Temperature, $T_i$ | 1 eV | 50 eV |
| Characteristic velocities | | |
| Alfvén Velocity, $v_A = B/(\mu_0 n_i m_i)$ | $3.38 \times 10^6$ m s$^{-1}$ | $1.25 \times 10^7$ m s$^{-1}$ |
| Electron Thermal Velocity, $v_{te} = (2T_e/m_e)^{1/2}$ | $1.19 \times 10^6$ m s$^{-1}$ | $4.17 \times 10^6$ m s$^{-1}$ |
| **Electron Thermal to Alfvén Velocity**, $v_{te}/v_A$ | **0.35** | **0.33** |
| Alfvén wave parameters | | |
| Parallel Wavelength, $\lambda_z$ | 2.82 m | 500 km |
| Perpendicular Wavelength, $\lambda_\perp$ | 0.105 m | 10 km |
| Wave Magnetic Field Amplitude $\delta B_\perp$ | 15 $\mu$T | 10 nT |
| Wave Perpendicular Electric Field Amplitude $E_\perp$ | 50 V m$^{-1}$ | 200 mV m$^{-1}$ |
| Wave Parallel Electric Field Field Amplitude $E_z$ | 0.15 V m$^{-1}$ | 2 mV m$^{-1}$ |

Supplementary Table 2: Comparison of plasma parameters. Parameters measured or estimated in the LAPD experiment compared to those predicted in the auroral acceleration region, where the key dimensionless parameter characterizing this region is the ratio of the electron thermal to Alfvén velocity, $v_{te}/v_A$ (bold).

For the comparison of the experiment to the corresponding position in the auroral acceleration region with $v_A/v_{te} = 3$ at $s \simeq 2.85$ $\mathrm{R_E}$, or an altitude $z \simeq 2.56$ $\mathrm{R_E}$, the resulting plasma parameters are given in Supplementary Table 2. At this position, we take a typical Alfvén wave amplitude of $\delta B_\perp = 10$ nT [32, 33, 34], which corresponds to a perpendicular electric field wave amplitude of $E_\perp \sim 200$ mV m$^{-1}$. For a perpendicular wavenumber $k_\perp \delta_e \sim 1$, the perpendicular wavelength is approximately $\lambda_\perp \sim 10$ km. The parallel wavelength is typically estimated to be a factor of 10 to 100 times longer than the perpendicular wavelength [31], and such a value is also consistent with the parallel wavelength that would be estimated by the condition of critical balance in strong Alfvénic turbulence [35]. Here we take the parallel wavelength to have a value of $L_\| = 500$ km. With the value of $L_\|/L_\perp \sim 50$ and $k_\perp \delta_e \sim 1$, the relation between for $E_\|/E_\perp$ given by Chaston (2006) [31] yields a value $E_z \sim 2$ mV m$^{-1}$.

# References

[1] Kletzing, C. A., Mozer, F. S. & Torbert, R. B. Electron temperature and density at high latitude. *J. Geophys. Res.* **103**, 14837–14846 (1998).

[2] Thuecks, D. J., Kletzing, C. A., Skiff, F., Bounds, S. R. & Vincena, S. Tests of collision operators using laboratory measurements of shear Alfvén wave dispersion and damping. *Physics of Plasmas* **16**, 052110 (2009).

[3] Kletzing, C. A., Thuecks, D. J., Skiff, F., Bounds, S. R. & Vincena, S. Measurements of Inertial Limit Alfvén Wave Dispersion for Finite Perpendicular Wave Number. *Physical Review Letters* **104**, 095001 (2010).

[4] Drake, D. J., Kletzing, C. A., Skiff, F., Howes, G. G. & Vincena, S. Design and use of an Elsässer probe for analysis of Alfvén wave fields according to wave direction. *Rev. Sci. Instrum.* **82**, 103505 (2011).

[5] Elsasser, W. M. The Hydromagnetic Equations. *Physical Review* **79**, 183–183 (1950).

[6] Arunasalam, V., Meservey, E. B., Gurnee, M. N. & Davidson, R. C. Absorption in a Hot Plasma near the Second Harmonic of the Electron Cyclotron Frequency. *Phys. Fluids* **11**, 1076–1084 (1968).

[7] Kirkwood, R., Hutchinson, I., Luckhardt, S. & Squire, J. Measurement of suprathermal electrons in tokamaks via electron cyclotron transmission. *Nuclear Fusion* **30**, 431 (1990). URL `http://stacks.iop.org/0029-5515/30/i=3/a=005`.

[8] Skiff, F., Boyd, D. A. & Colborn, J. A. Measurements of electron parallel-momentum distributions using cyclotron wave transmission*. *Physics of Fluids B* **5**, 2445–2450 (1993).

[9] Skiff, F., Boyd, D. A. & Colborn, J. A. Measurements of electron dynamics during lower hybrid current drive. *Plasma Physics and Controlled Fusion* **36**, 1371–1379 (1994).

[10] Thuecks, D. J., Skiff, F. & Kletzing, C. A. Measurements of parallel electron velocity distributions using whistler wave absorption. *Review of Scientific Instruments* **83**, 083503 (2012).

[11] Schroeder, J. W. R. *et al.* Linear theory and measurements of electron oscillations in an inertial Alfvén wave. *Phys. Plasmas* **24**, 032902 (2017).

[12] Schroeder, J. W. R. *et al.* Direct measurement of electron sloshing of an inertial Alfvén wave. *Geophys. Res. Lett.* **43**, 4701–4707 (2016).

[13] Klein, K. G. & Howes, G. G. Measuring Collisionless Damping in Heliospheric Plasmas using Field-Particle Correlations. *Astrophys. J. Lett.* **826**, L30 (2016). `1607.01738`.

[14] Howes, G. G., Klein, K. G. & Li, T. C. Diagnosing collisionless energy transfer using field-particle correlations: Vlasov-Poisson plasmas. *J. Plasma Phys.* **83**, 705830102 (2017).

[15] Klein, K. G., Howes, G. G. & TenBarge, J. M. Diagnosing collisionless enegy transfer using field-particle correlations: gyrokinetic turbulence. *J. Plasma Phys.* **83**, 535830401 (2017). `1705.06385`.

[16] Chen, C. H. K., Klein, K. G. & Howes, G. G. Evidence for electron Landau damping in space plasma turbulence. *Nature Comm.* **10**, 740 (2019). URL `https://doi.org/10.1038/s41467-019-08435-3`.

[17] Numata, R., Howes, G. G., Tatsuno, T., Barnes, M. & Dorland, W. AstroGK: Astrophysical gyrokinetics code. *J. Comp. Phys.* **229**, 9347 (2010). `1004.0279`.

[18] Abel, I. G., Barnes, M., Cowley, S. C., Dorland, W. & Schekochihin, A. A. Linearized model Fokker-Planck collision operators for gyrokinetic simulations. I. Theory. *Phys. Plasmas* **15**, 122509 (2008). `0808.1300`.

[19] Barnes, M. *et al.* Linearized model Fokker-Planck collision operators for gyrokinetic simulations. II. Numerical implementation and tests. *Phys. Plasmas* **16**, 072107 (2009).

[20] Howes, G. G. *et al.* Astrophysical Gyrokinetics: Basic Equations and Linear Theory. *Astrophys. J.* **651**, 590–614 (2006). `astro-ph/0511812`.

[21] Nielson, K. D., Howes, G. G. & Dorland, W. Alfvén wave collisions, the fundamental building block of plasma turbulence. II. Numerical solution. *Phys. Plasmas* **20**, 072303 (2013). `1306.1456`.

[22] Howes, G. G., McCubbin, A. J. & Klein, K. G. Spatially localized particle energization by Landau damping in current sheets produced by strong Alfvén wave collisions. *J. Plasma Phys.* **84**, 905840105 (2018). `1708.00757`.

[23] Gurnett, D. A. & Bhattacharjee, A. *Introduction to Plasma Physics* (Cambridge, UK: Cambridge University Press, 2005).

[24] Kletzing, C. A. Electron acceleration by kinetic Alfvén waves. *J. Geophys. Res.* **99**, 11095–11104 (1994).

[25] Kletzing, C. A. & Hu, S. Alfvén wave generated electron time dispersion. *Geophys. Res. Lett.* **28**, 693–696 (2001).

[26] Stix, T. H. *Waves in plasmas* (New York: American Institute of Physics, 1992).

[27] O'Neil, T. Collisionless Damping of Nonlinear Plasma Oscillations. *Phys. Fluids* **8**, 2255–2262 (1965).

[28] Stasiewicz, K. *et al.* Small Scale Alfvénic Structure in the Aurora. *Space Sci. Rev.* **92**, 423–533 (2000).

[29] Keiling, A. Alfvén Waves and Their Roles in the Dynamics of the Earth's Magnetotail: A Review. *Space Sci. Rev.* **142**, 73–156 (2009).

[30] Kletzing, C. A. & Torbert, R. B. Electron time dispersion. *J. Geophys. Res.* **99**, 2159–2172 (1994).

[31] Chaston, C. C. ULF Waves and Auroral Electrons. In Takahashi, K., Chi, P. J., Denton, R. E. & Lysak, R. L. (eds.) *Magnetospheric ULF Waves: Synthesis and New Directions*, vol. 169 of *Washington DC American Geophysical Union Geophysical Monograph Series*, 239 (2006).

[32] Keiling, A. *et al.* Correlation of Alfvén wave Poynting flux in the plasma sheet at 4-7 $R_E$ with ionospheric electron energy flux. *Journal of Geophysical Research (Space Physics)* **107**, 1132 (2002).

[33] Wygant, J. R. *et al.* Evidence for kinetic Alfvén waves and parallel electron energization at 4-6 $R_E$ altitudes in the plasma sheet boundary layer. *Journal of Geophysical Research (Space Physics)* **107**, 1201 (2002).

[34] Schriver, D. *et al.* FAST/Polar conjunction study of field-aligned auroral acceleration and corresponding magnetotail drivers. *Journal of Geophysical Research (Space Physics)* **108**, 8020 (2003).

[35] Goldreich, P. & Sridhar, S. Toward a Theory of Interstellar Turbulence II. Strong Alfvénic Turbulence. *Astrophys. J.* **438**, 763–775 (1995).
